# PARK2 promotes mitochondrial pathway of apoptosis and antimicrotubule drugs chemosensitivity *via* degradation of phospho-BCL-2

**DOI:** 10.7150/thno.47044

**Published:** 2020-08-08

**Authors:** Hengxing Chen, Yun Li, Yu Li, Zhen Chen, Limin Xie, Wenjia Li, Yuanxin Zhu, Hong Xue, H. Phillip Koeffler, Wenjing Wu, Kaishun Hu, Dong Yin

**Affiliations:** 1 Guangdong Provincial Key Laboratory of Malignant Tumor Epigenetics and Gene Regulation, Medical Research Center, Sun Yat-Sen Memorial Hospital, Sun Yat-Sen University, Guangzhou, China.; 2 Division of Life Science, Applied Genomics Centre and Centre for Statistical Science, Hong Kong University of Science and Technology, Clear Water Bay, Kowloon, Hong Kong, China.; 3 Cancer Science Institute of Singapore, National University of Singapore, Singapore.; 4 Division of Hematology/Oncology, Cedars-Sinai Medical Center, University of California Los Angeles School of Medicine, Los Angeles, CA, USA.; 5 Department of Breast Oncology, Sun Yat-Sen Memorial Hospital, Sun Yat-Sen University, Guangzhou, 510120, China.

**Keywords:** PARK2, antimicrotubule drug, docetaxel, phospho-BCL-2, breast cancer

## Abstract

**Rationale:** Neoadjuvant chemotherapy has become the standard treatment of locally advanced breast cancer. Antimicrotubule drugs and DNA-damaging drugs are the most popular medicines used for neoadjuvant chemotherapy. However, we are unable to predict which chemotherapeutic drug will benefit to an individual patient. PARK2 as a tumor suppressor in breast cancer has been reported. While the role of PARK2 in chemotherapy response remains unknown. In this study, we explore the impact of PARK2 on chemosensitivity in breast cancer.

**Methods:** PARK2 expression in breast cancer patients with different neoadjuvant chemotherapeutic regimens was studied using immunohistochemistry. Data was correlated to disease-free survival (DFS), overall survival and pathologic complete response (pCR). The functional roles of PARK2 were demonstrated by a series of *in vitro* and *in vivo* experiments. Including mass spectrometry, Co-immunoprecipitation, isolation of subcellular fractionation, fluorescence microscopy, *in vivo* ubiquitination assay and luciferase analyses.

**Results:** Highly expressed PARK2 predicted better response to antimicrotubule drugs-containing regimen associated with higher rate of pathologic complete response (pCR). In contrast, PARK2 expression did not predict response to the DNA-damaging drugs regimen. Following antimicrotubule drugs treatment, levels of PARK2 was upregulated due to the repression of STAT3-mediated transcriptional inhibition of PARK2. Moreover, overexpression of PARK2 specifically rendered cells more sensitive to antimicrotubule drugs, but not to DNA-damaging drugs. Depletion of PARK2 enhanced resistance to antimicrotubule drugs. Mechanistically, PARK2 markedly activated the mitochondrial pathway of apoptosis after exposure to antimicrotubule drugs. This occurred through downregulating the antiapoptotic protein, phospho-BCL-2. BCL-2 phosphorylation can be specifically induced by antimicrotubule drugs, whereas DNA-damaging drugs do not. Notably, PARK2 interacted with phospho-BCL-2 (Ser70) and promoted ubiquitination of BCL-2 in an E3 ligase-dependent manner. Hence, PARK2 significantly enhanced the chemosensitivity of antimicrotubule drugs both *in vitro and in vivo*, while loss-of-function PARK2 mutants did not.

**Conclusions:** Our findings explained why PARK2 selectively confers chemosensitivity to antimicrotubule drugs, but not to DNA-damaging drugs. In addition, we identified PARK2 as a novel mediator of antimicrotubule drugs sensitivity, which can predict response of breast cancer patients to antimicrotubule drugs-containing regime.

## Introduction

Breast cancer is one of the most leading worldwide causes of cancer morbidity and mortality in women. Neoadjuvant chemotherapy (NAC) for advanced breast cancer is considered one of the most crucial treatments for reducing mortality 1, 2. Thus, NAC become the standard treatment of locally advanced breast cancer, which can downstage the disease and improve surgical option 3. Antimicrotubule drugs and DNA-damaging drugs are commonly used NAC drugs 1. Antimicrotubule drugs, such as taxane and vinca alkaloid suppress microtubule dynamics, lead to G2/M cell cycle arrest, and trigger apoptosis through the mitochondrial pathway 4-6. DNA-damaging drugs, such as alkylator and anthracyclines cause cell death through directly destroying DNA. Sadly, only a small percentage of patients receiving NAC achieve a complete response, while the others suffer from the side effects of chemotherapy without benefit from it. Ideally, patients who would benefit from a particular chemotherapy regimen will be identified, protecting the remaining from side effects. Therefore, the developments of predictive biomarkers are urgently needed to guide the use of NAC regimen.

Antimicrotubule drugs induce cellular apoptosis. However, antimicrotubule drugs also trigger stress responses that inhibit apoptosis and promote emergence of an acquired treatment-resistant phenotype. Antiapoptotic protein, BCL-2, has a key roles in these stress responses. BCL2 protein inhibits apoptosis by binding to BAX protein and inhibiting the activation of the latter 7-9. Antimicrotubule drugs induce phosphorylation of BCL-2 on its serine (Ser) residues, Ser70. Phospho-BCL-2 (S70) greatly enhances BCL-2's antiapoptotic function. Briefly, mono- or multisite phosphorylation can enhance BCL-2's survival activity 10. JNK induced-BCL-2 phosphorylation at Ser70 prolongs cell survival following stress stimuli 11. BCL-2 phosphorylation is required for full BCL-2 death-suppressing signaling activity 12. Phospho-BCL-2 (S70) indeed enhanced BCL-2's antiapoptotic activity in MCF-7 cells (Figure S4E-G).

Loss-of-function mutants of PARK2 have been identified as one of the most common causes of recessive familial early onset Parkinsonism 13, 14. PARK2 is mutated and/or deleted in a variety of human malignancies 15, 16. It is a tumor suppressor in a wide variety of human cancers including breast cancer, whose loss significantly increases tumorigenesis 17, 18. PARK2 gene encodes a well-conserved RING-in-between-RING (RBR) type E3 ubiquitin ligase Parkin, which targets specific substrates through the ubiquitin-proteasome system for degradation 19. PARK2 is also involved in the regulation of various cellular processes, including stress response and mitochondrial biogenesis 20.

In this study, we report a significant correlation between PARK2 expression and chemosensitivity to antimicrotubule drugs-containing regimen in breast cancer patients. Among patients who received antimicrotubule drugs, higher expression of PARK2 showed better disease-free survival (DFS), overall survival and higher rate of pathologic complete response (pCR). In contrast, no significant differences were noted between PARK2 expression and survival in those who received DNA-damaging drugs (without antimicrotubule drugs). Moreover, overexpression of PARK2 renders cells more sensitive to antimicrotubule drugs in breast cancer cell lines, which is not observed in a range of DNA-damaging drugs. In addition, our studies reveal that antimicrotubule drug stimulates PARK2 expression via downregulating STAT3. PARK2 markedly activates the antimicrotubule drug-induced mitochondrial pathway of apoptosis by degrading phospho-BCL-2 (Ser70). Taken together, our study shows that PARK2 enhances chemosensitivity of breast cancer cells to antimicrotubule drugs and provides a predictive biomarker for antimicrotubule drugs-containing regimen.

## Materials and Methods

### Reagents and antibodies

For details, please refer to Table S2.

### Plasmids

The pEGFP-parkin WT, pRK5-HA-Ubiquitin-WT, and pRK5-HA-Ubiquitin-KO were obtained from Addgene (Plasmid #45875, Plasmid #17608, and Plasmid #17603). Full-length cDNA coding for PARK2 was obtained from the cDNA of human HEK293 cells using PCR and was then cloned into pLVX-DsRed-Monomer-N1 vector also having a FLAG-tag sequence. Full-length cDNA coding for BCL-2 was obtained from the cDNA of human MCF-7 cells using PCR and was cloned into pLVX-DsRed-Monomer-N1 vector also having a MYC-tag sequence. Point mutations were introduced by site-directed mutagenesis. SFB-PARK2 was first subcloned into pDONR201 entry vectors and transferred into destination vector with the indicated SFB tag using Gateway Technology (Invitrogen, Camarillo, CA, USA). Doxycycline-inducible PARK2 constructs were cloned from human HEK293 cDNA and inserted into pRL-TetON-EGFP vector using EcoR I and Sali sites. All shRNA constructs for PARK2 were made using pLKO.1 backbone employing Age I and EcoR I sites. For sequences of shRNAs and siRNAs, please refer to Table S2.

### Western blotting and co-immunoprecipitation

RIPA buffer (150 mM NaCl, 50 mM Tris-HCl [pH 8.0], 0.5% Nonidet P-40, 5 mM EDTA and a protease and phosphatase inhibitor cocktail (Bimake, Houston, TX, USA) were used to lyse cells. SDS-PAGE resolved the clarified lysates. Samples were transferred to PVDF membranes for Western blots and detected by ECL detection reagents (Beyotime, Shanghai, China). For immunoprecipitation, supernatants were first incubated with anti-FLAG agarose overnight at 4°C. Subsequently, pellets were washed three times with NETN buffer. To examine endogenous interactions, cell lysates were divided into two parts, incubated with either anti-IgG, anti-BCL-2 or anti-PARK2 for 2 h, and then incubated with protein G agarose (Life Technologies, Waltham, MA, USA) overnight. Beads were then washed three times with NETN buffer; and the samples were resolved on SDS-PAGE.

### *In vivo* ubiquitination assay

The process was carried out as previously described 21. Briefly, MCF-7 cells were transfected with either pEGFP-parkin WT, pRK5-HA-Ubiquitin-WT or pRK5-HA-Ubiquitin-KO plasmid for 18 h. Cells were either treated or not treated with docetaxel (1 nM) for 24 h. Cells were then cultured with MG132 (10 mM) followed by immunoprecipitation (IP) analysis with BCL-2 antibody. Endogenous BCL-2 was immunoprecipitated using anti-BCL-2 antibody for 12 h at 4°C. Anti-HA antibody was used to detect poly-ubiquitinated BCL-2.

MDA-MB-134-VI cells were stably either deleted endogenous PARK2 or NC (control). These cells were transfected with pRK5-HA-Ubiquitin-WT for 18 h, and were either treated or not treated with docetaxel (2 nM) for 24 h. Cells were then treated with MG132 (10 mM) for 4 h and followed by immunoprecipitation (IP) analysis with BCL-2 antibody.

### Cytotoxicity assay

Cells were seeded in 96-well plates at 1,000 cells/well in culture media with a variety of chemotherapy drugs for 48 h. Cell viability was analyzed using the MTT as previously described 22. Briefly, 10 μL of 5 mg/mL MTT solution was added to each well. The wells were incubated in a humidified chamber for 4 h and terminated by addition of 100 μL of 10% sodium dodecyl sulfate (SDS). The samples were mixed thoroughly until the formazan was dissolved and then detected by 570 nm absorbance.

### Patients and tissue samples

205 paraffin-embedded breast cancer specimens, which were pathologically and clinically diagnosed at the Sun Yat-sen Memorial Hospital, were utilized. Patients' consent and approval from the ethical board of Sun Yat-sen University were obtained for research in use of clinical materials. Clinical information on these patients, including tumor size, chemotherapy regimens, survival situation, etc., was obtained from past medical records and recent follow-up records (Table S1). All the clinical diagnostic information and clinico-pathological variables were classified according to the American Joint Committee. Pathologic complete remission (pCR) was defined as disappearance of invasive tumor lesion in the surgically removed breast and axillary lymph nodes after chemotherapy.

### Immunohistochemical staining and scoring

IHC staining was performed as described previously. The histopathological features of the stained tumor were evaluated by two researchers who were unaware of the patient's clinical characteristics. Intensity of PARK2 immunostaining of breast cancer tissues was scored as negative (0), weak (1), medium (2) or strong (3). The extent of staining, defined as the percent positive staining cells, was scored as 1 (≤ 10%), 2 (11-50%), 3 (51-75%) or 4 (>75%). Staining index (SI) was calculated as follows: SI = staining intensity × proportion of positively stained cells. Low and high expression were defined as scores of < 7 and ≥ 7, respectively.

### Mass spectrometry (label-free protein quantification)

4 plates of MCF-7 cells which stably expressed SFB-tagged PARK2 were divided into two groups, either treated or not treated with docetaxel (1 nm) for 24 h, respectively. Followed by cells lysed with NETN buffer for 20 min. Supernatant was removed via centrifugation at 15,000 g to remove debris and then incubated with streptavidin-conjugated beads (Amersham Biosciences, Franklin Lakes, NJ, USA) for 12 h at 4°C. Beads were washed three times with NETN buffer, followed by incubated with S-protein beads (Sigma, St. Louis, MO, USA) for 6 h at 4°C. Beads were washed three times with NETN buffer followed by digestion of lysed cells. Peptides were analyzed by LC-MS/MS. Label-free quantifycation was carried out at the peptide level. These peptides were identified from individual second dimension fractions which were assembled into a master list. Median of the computed peptide ratios represents relative quantity of the protein in the two samples. Quantitative proteomics used a label-free method to measure relative protein abundance.

### Bioinformatics and data analysis

Copy-number data of breast cancer were collected from The Cancer Genome Atlas (TCGA) via cBio Cancer Genomics Portal and analyzed with IGV software. Copy-number data of breast cancer cell lines were obtained from the Cancer Cell Line Encyclopedia database. Correlations between the overall survival of breast cancer patients and the expression of PARK2 by their tumor were analyzed via KM plotter. Analysis of sensitivity of PARK2 gene to chemotherapy drugs were collected from the ONCOMINE database. Correlation between the BCL-2 protein level and PARK2 mRNA expression was analyzed by The Cancer Genome Atlas and The Human Protein Atlas database. Somatic PARK2 mutations in cancer were obtained from cBio Cancer Genomics Portal.

### Statistical analysis

GraphPad Prism version 7 was used to analyze the data. Each group was analyzed three times. All experiments subjected to statistical analysis were repeated at least three times. Results are presented as mean ± standard error of mean (SEM) and comparisons were made using T-test statistical analysis (non-parametric).

The rest of the Materials and Methods are shown in the Supplementary Information.

## Results

### PARK2 expression predicts clinical outcome of antimicrotubule drugs-containing regimen

**Figure 1:**
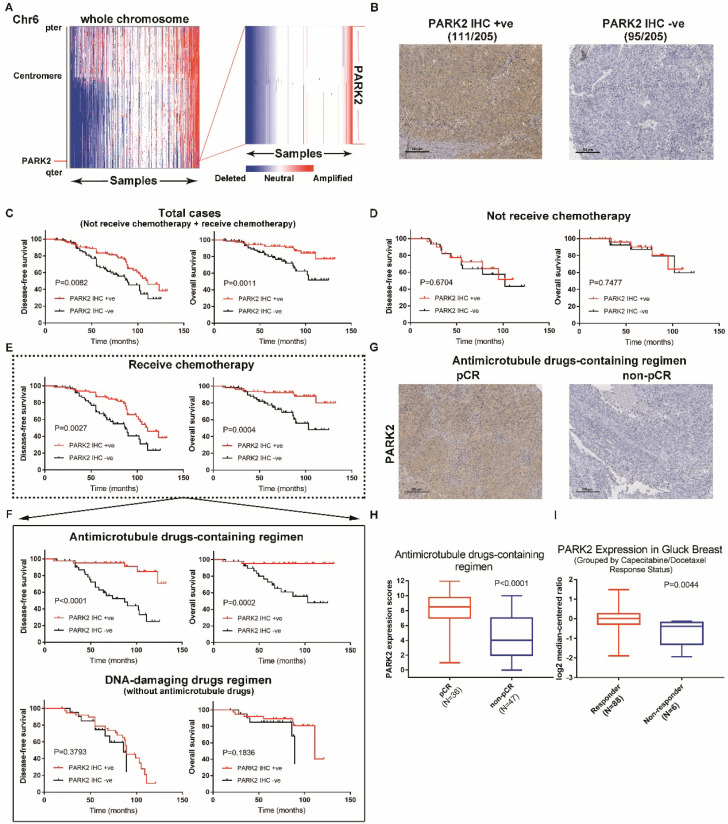
** PARK2 predicts clinical outcome of antimicrotubule drugs-containing regimen. A.** IGV plots showed that PARK2 gene (located on chromosome 6) is often deleted in breast cancer (436 of 1,283 breast cancer samples). **B.** Representative IHC staining intensities of PARK2 protein expression in breast cancer tissues. **C.** Higher PARK2 expression was associated with better DFS and overall survival of breast cancer patients. High expression (n=111), Low expression (n=94). **D.** Kaplan-Meier estimates of DFS and overall survival of the breast cancer who did not receive chemotherapy. High expression (n=31), Low expression (n=31). **E.** Kaplan-Meier estimates of DFS and overall survival of the breast cancers who received chemotherapy. High expression (n=80), Low expression (n=63). **F.** Above graph: Kaplan-Meier estimates of DFS and overall survival of the breast cancer who received antimicrotubule drugs-containing regimen. High expression (n=42), Low expression (n=41). Below graph. Kaplan-Meier estimates of DFS and overall survival of the breast cancer who received DNA-damaging drugs regimen (without antimicrotubule drugs). High expression (n=38), Low expression (n=22). **G.** Representative images from immunohistochemical staining of PARK2 in tumors from four cases of breast cancer who receive antimicrotubule drugs with different pathologic complete response (pCR). **H.** Expression levels of PARK2 in breast cancer patients, who received antimicrotubule drugs-containing regimen. **I.** Upregulation of PARK2 mRNA expression in the Capecitabine/Docetaxel responding group compared with the non-responding group.

PARK2 gene is located on chromosome 6, it is often mutated and/or deleted in cancer. Copy number loss of PARK2 gene is the most frequent somatic alteration 15, 16. Therefore, we examined PARK2 deletion in The Cancer Genome Atlas (TCGA), assembling data from 1,283 primary breast tumors. A total of 34% of breast cancer patients exhibited a deletion of PARK2 (Figure 1A). Copy number loss of PARK2 also occurred in 53% (32/60) of breast cancer cell lines (Figure S1A). Next, the prognostic value of PARK2 expression in breast cancer was analyzed. Higher expression of PARK2 was significantly correlated with better survival of patients with breast cancer (Kaplan-Meier plotter database) (Figure S1B). We classified breast cancer into different molecular subtypes, including basal, HER2+, luminal A, and luminal B. Statistically, higher PARK2 expression was strongly correlated with better survival in patients with basal, HER2+, and luminal A subtypes. Although the trend was the same, this correlation within luminal B subtype was not statistically significant (Figure S1C). Furthermore, we divided the data set into two groups: those who received chemotherapy, and those who did not. Interestingly, for patients who did not receive chemotherapy, no difference occurred in survival between patients with either high or low expression of PARK2. However, among patients who received chemotherapy, higher PARK2 expression was positively correlated with better survival (Figure S1D). Together, these finding suggest that prominent PARK2 levels enhance chemotherapy in breast cancer.

Breast cancer patients received neoadjuvant chemotherapy including either antimicrotubule drugs or DNA-damaging drugs, but the details of chemotherapy were not included in Kaplan Meier plotter database. Indicators to determine the efficacy of chemotherapy include disease-free survival (DFS), overall survival and pathologic complete response (pCR). Thus, we collected 205 tumor tissues samples from breast cancer patients and analyzed the expression of PARK2. Immunohistochemical analysis showed that 95 were PARK2-negative (-), 111 were PARK2-positive (+) (Figure 1B). Highly expressed PARK2 showed significantly better disease-free survival (DFS) and overall survival than those with tumors with low PARK2 expression (Figure 1C). A total of 62 patients did not receive neoadjuvant chemotherapy. No significant differences accured in their DFS and overall survival regardless of the amounts of PARK2 in the tumors (Figure 1D). In contrast, for the remaining 143 patients, who receive neoadjuvant chemotherapy, higher expression of PARK2 in tumors was associated with better DFS and overall survival (Figure 1E). This is consistent with the previous database analysis. Furthermore, we divided the patients who received neoadjuvant 'chemotherapy into two groups: those who received antimicrotubule drugs-containing regimen, and those who received DNA-damaging drugs regimen (without antimicrotubule drugs). Among patients who received DNA-damaging drugs regimen (without antimicrotubule drugs), PARK2 expression was not associated with DFS and overall survival (Figure 1F). However, for patients who received antimicrotubule drugs-containing regimen treatment, higher expression of PARK2 was significantly correlated with better DFS and overall survival (Figure 1F). Among these patients, PARK2 level in the pathologic complete response (pCR) group was substantially higher than that in the non-pathologic complete response (non-pCR) group (Figure 1G&H). Moreover, we found that PARK2 mRNA level in the Capecitabine/Docetaxel responding group was substantially higher than that in the non-responding group (ONCOMINE database) (Figure 1I).

Collectively, these findings suggested that PARK2 expression could predict the clinical outcome of antimicrotubule drugs-containing regimen therapy for breast cancer.

### PARK2 regulates antimicrotubule drugs sensitivity in breast cancer cells

Chemotherapy drugs used in breast cancer include docetaxel, paclitaxel, adriamycin, cyclophosphamide, 5-fluorouracil and vinorelbine. These can be divided into two categories: antimicrotubule agents (Docetaxel, paclitaxel and vinorelbine) and DNA-damaging agents (Adriamycin, cyclophosphamide and 5-fluorouracil) 4.

**Figure 2:**
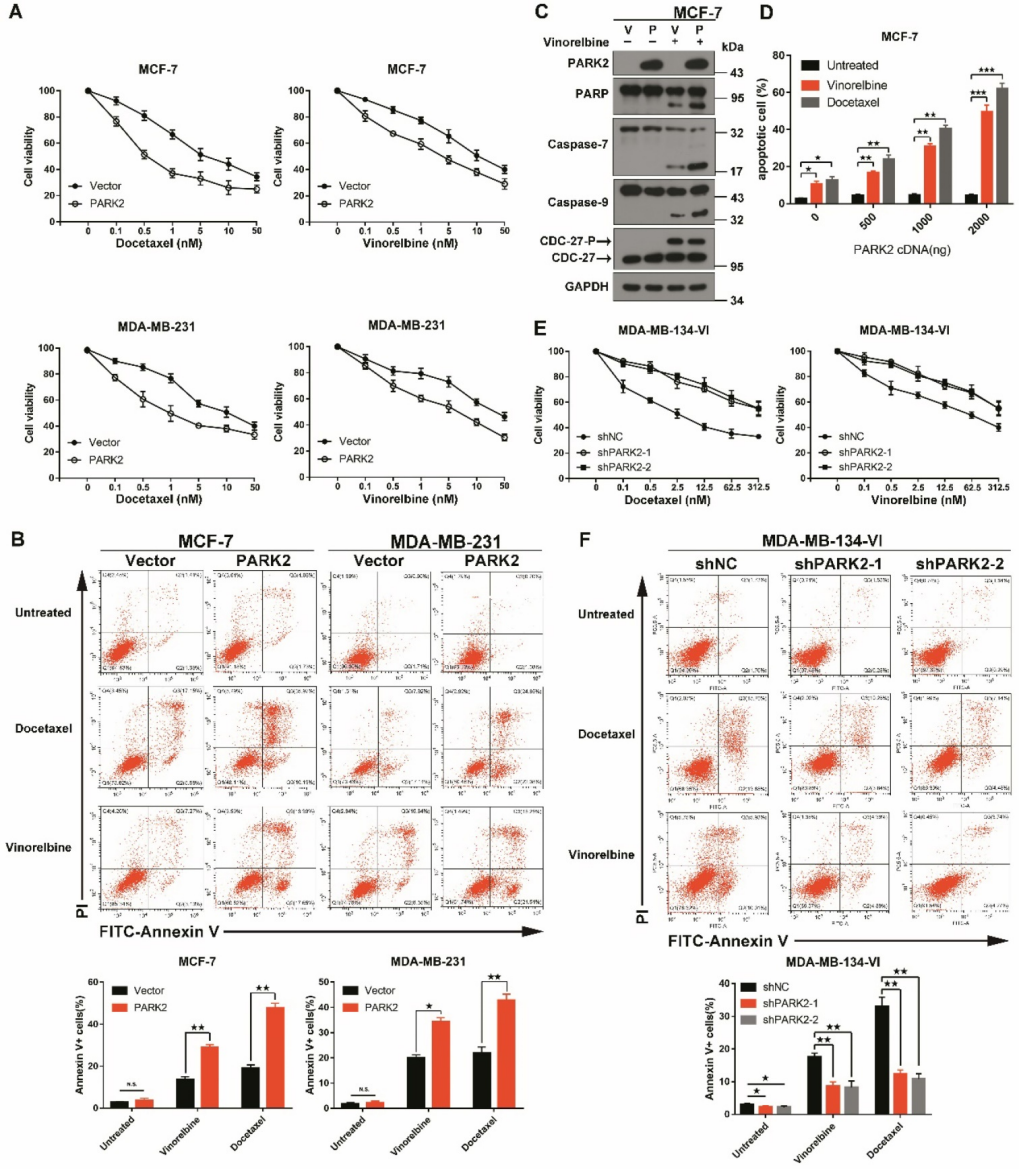
** PARK2 regulates antimicrotubule drugs sensitivity in breast cancer cells. A.** Cytotoxicity assays showed the sensitivity to docetaxel and vinorelbine. Breast cancer lines stably expressing either ectopic wild-type PARK2 or vector were treated with the indicated compounds for 48 h. **B.** PARK2 overexpression increases apoptosis of breast cancer cells after either docetaxel or vinorelbine treatment for 48 h. Representative FACS profiles are shown, on which the population of cells in the quadrant (Annexin V± DAPI) represents apoptotic cells. **C.** MCF-7 cells stably expressing either vector control (V) or PARK2 (P) were treated with vinorelbine (1 nM) for 48 h. Cell lysates were immunoblotted for caspases and caspase substrates. Phosphorylation of CDC27 is an indicator of mitotic arrest and we used it to indicate effectiveness of antimicrotubule drugs. CDC27-P, phosphorylated CDC27. **D.** MCF-7 cells were transfected with empty vector along with the indicated amount of PARK2 cDNA for 18 h. After transfection, cells were either treated or not with either docetaxel (1 nM) or vinorelbine (1 nM) for 36 h; cell death was analyzed by flow cytometry. **E.** Cytotoxicity assay measuring the sensitivity to docetaxel and vinorelbine. ShNC and shPARK2 MDA-MB-134-VI cells treated with the indicated compounds for 48 h. **F.** Silencing PARK2 (shPARK2) decreased cell apoptosis of breast cancer cells after either docetaxel or vinorelbine treatment for 48 h. Data show mean ± s.d. N = 3. *P < 0.05, **P < 0.01, ***P < 0.001.

To confirm that overexpression of PARK2 only contributes to antimicrotubule drug sensitivity; we ectopically expressed PARK2 in MCF-7 and MDA-MB-231 cells. Cytotoxicity assays were done, and because of close similarity of docetaxel and paclitaxel, only the former was tested. Forced expression of PARK2 rendered breast cancer cells more sensitive to docetaxel and vinorelbine in both MCF-7 and MDA-MB-231 cells (Figure 2A and S2A), but overexpression of PARK2 did not change the sensitivity of DNA-damaging drugs (Figure S2B). Flow cytometry showed that PARK2 overexpressing cells (MCF-7, MDA-MB-231) led to marked increase in apoptosis when exposed to either docetaxel or vinorelbine (Figure 2B). This was consistent with a significantly higher apoptotic response, as indicated by the proteolysis of multiple caspase substrates (Figure 2C and 4E). Moreover, the higher PARK2 expression was paralleled by the greater sensitivity of docetaxel and vinorelbine in MCF-7 cells (Figure 2D). Furthermore, shRNA silencing of endogenous PARK2 resulted in significantly decreased sensitivity to docetaxel and vinorelbine in MDA-MB-134-VI cells (MDA-MB-134-VI: inherently high expression of PARK2) (Figure 2E and S2C). Flow cytometry showed that silencing of endogenous PARK2 reduced cell apoptosis after either docetaxel or vinorelbine exposure (Figure 2F).

Taken together, these results suggested that PARK2 conferred sensitivity to antimicrotubule drugs in breast cancer cells.

### Antimicrotubule drugs stimulates PARK2 expression

**Figure 3:**
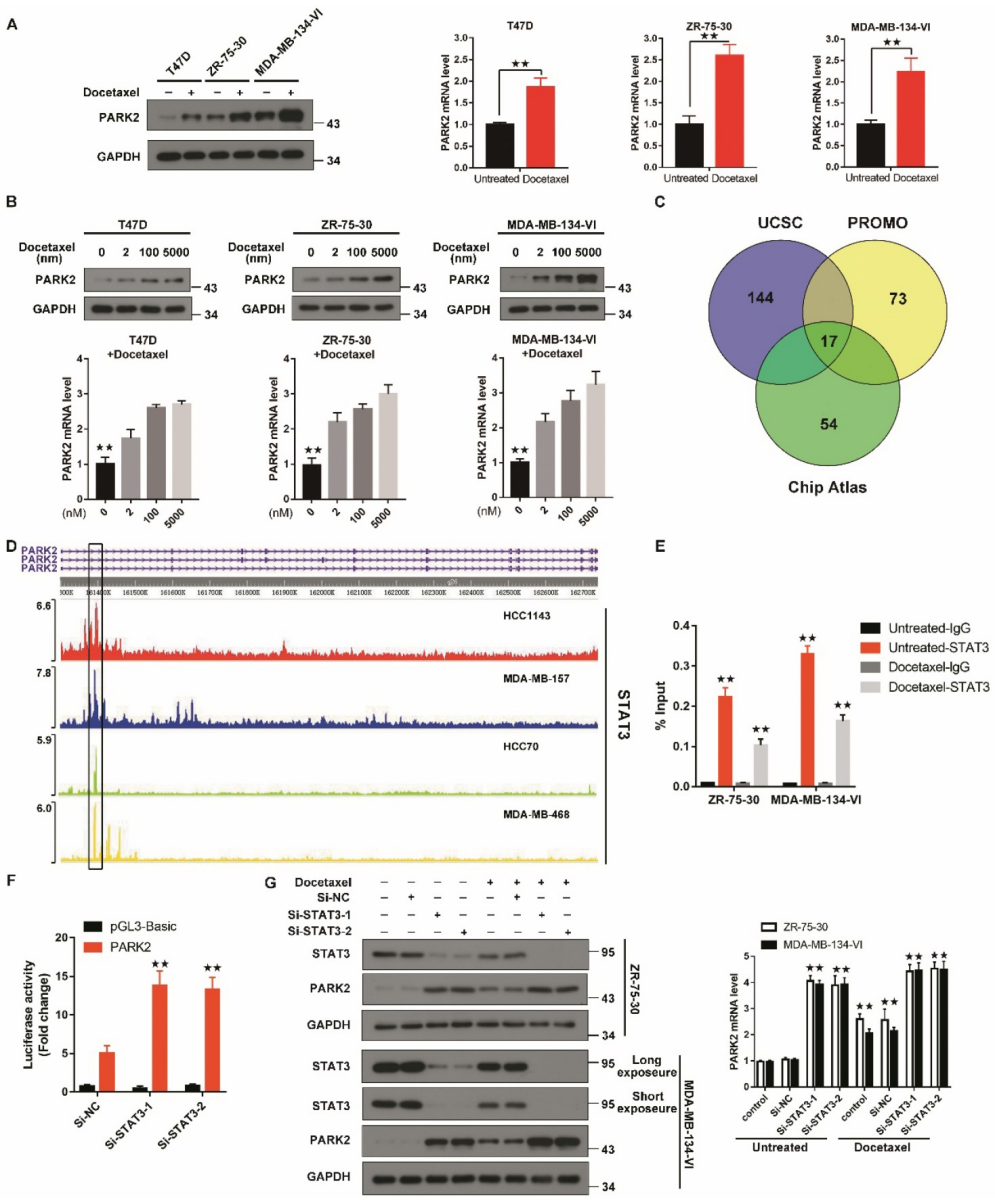
** Antimicrotubule drugs stimulate PARK2 expression. A&B.** Q-PCR and western blot confirmed that the mRNA and protein expression of PARK2 after docetaxel treatment. **C.** Prediction of TFs of PARK2 by overlaying ChIP-seq data from UCSC, Chip-Atlas and PROMO. **D.** Chip-seq profiles for STAT3 at PARK2 locus in breast cancer cell lines. **E.** Docetaxel treatment reduces enrichment of STAT3 on PARK2 promoter. **F.** Luciferase assay showing that STAT3 knockdown increased PARK2 promoter activity in MDA-MB-134-VI cells. **G.** Docetaxel induced downregulation of STAT3 and upregulation PARK2 expression, whereas silencing STAT3 increased PARK2 levels in both breast cancer cell lines. Data show mean ± s.d. N = 3. *P < 0.05, **P < 0.01.

To evaluate changes in PARK2 expression after antimicrotubule drugs treatment, qPCR and western blot were performed. Both mRNA and protein expression of PARK2 were significantly increased in T47D, ZR-75-30 and MDA-MB-134-VI cells when exposed to docetaxel (Figure 3A). Docetaxel also induced PARK2 expression in a dose-dependent manner in T47D, ZR-75-30 and MDA-MB-134-VI cells (Figure 3B).

To understand how PARK2 levels are upregulated by antimicrotubule treatment in breast cancer cells, we predicted putative transcription factors (TF) by overlaying the ChIP-seq data from the ChIP-Atlas database, the UCSC Genome Browser and the virtual laboratory of PROMO (Figure 3C). Intersection of data from the three databases showed seventeen TFs. Among these TFs, STAT3 protein has been reported to be decreased in response to antimicrotubule drugs treatment 23-25, whereas STAT3 is frequently enriched at the PARK2 promoter (Figure 3D). Indeed, Chip assay showed that STAT3 was enriched at the PARK2 promoter. After docetaxel treatment, the amount of STAT3 enriched on the PARK2 promoter was significantly reduced (Figure 3E). Luciferase assay showing that STAT3 knockdown increased PARK2 promoter activity in MDA-MB-134-VI cells (Figure 3F). We also found that STAT3 knockdown increased the PARK2 levels in ZR-75-30 and MDA-MB-134-VI cells (Figure 3G), whereas STAT3 overexpression downregulated the PARK2 levels in both cell lines (Figure S2D).

### PARK2 activates the mitochondrial pathway of apoptosis after exposure to antimicrotubule drugs

Antimicrotubule drugs induce cells to enter the mitochondrial apoptotic pathway 5, 26. Antimicrotubule drugs activate the pro-apoptotic protein, BAX, which forms oligomers. These activated BAX dimers translocate to the mitochondria and induce release of cytochrome C into the cytoplasm, resulting in proteolytic caspase activation followed by DNA fragmentation, and ultimately lead to cell death 7, 27.

**Figure 4:**
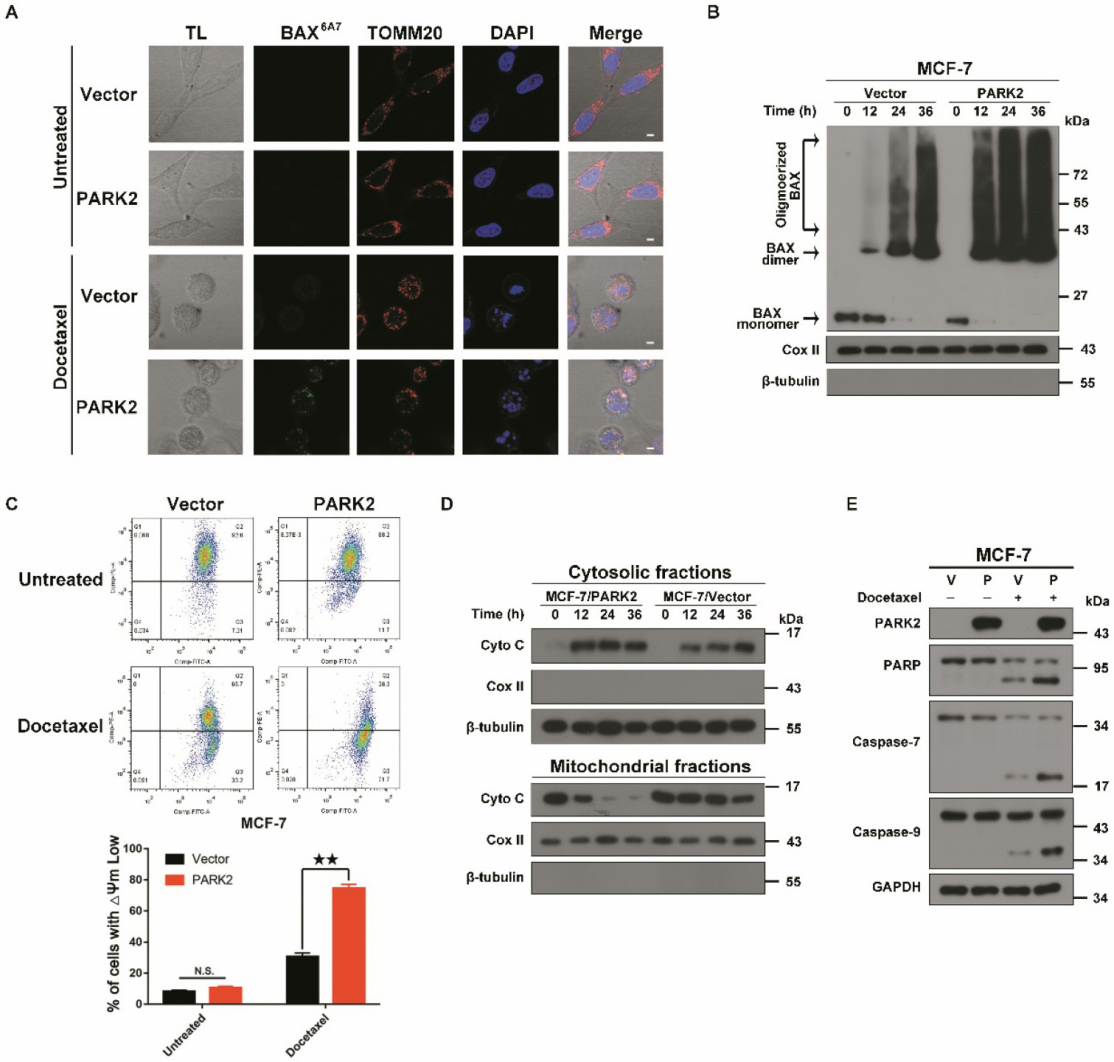
** PARK2 causes apoptosis by activating mitochondrial pathway induced by antimicrotubule drugs. A.** MCF-7 cells stably expressing either ectopic wild-type PARK2 or vector (control) were cultures either with or without docetaxel (1 nM) for 24 h. MCF-7 cells were co-immunostained with TOMM20 antibody (red; anti-TOMM20), activated form of BAX antibody (green; anti-BAX 6A7) and nuclei (blue; DAPI). Cells were analyzed by confocal microscopy. Scale bars represent 5 µm. **B.** MCF-7 cells stably expressing either ectopic wild-type PARK2 or vector control were treated with docetaxel (1 nM) for 0, 12, 24 and 36 h. After docetaxel stimulation, mitochondria were fractionated. In order to crosslink the BAX oligomerized proteins, mitochondria fractions were treated with a sulph-hydryl-reactive crosslinker [bis-maleimidohexane (BMH)]. Samples were separated on a 4-12% gradient NuPAGE gel. Blot were probed with anti-BAX, anti-CoxII and anti-β-tubulin. **C.** Overexpression of PARK2 reduced mitochondrial membrane potential after docetaxel treatment. Representative FACS spectrum is shown in which the population of cells in the Q3 quadrant (right, bottom) represented the decrease of mitochondrial membrane potential. **D.** PARK2 promoted the release of cytochrome C from mitochondria to the cytosol. Isolation of cytosol and mitochondrial fractions and detection with anti-cytochrome C. For internal control, anti-Cox IV or anti-α-tubulin was used to probe the mitochondrial and cytoplasmic fractions, respectively. **E.** MCF-7 cells stably expressing either ectopic wild-type PARK2 or vector (control) were treated either with or without docetaxel (1 nM) for 48 h. Cell lysates were immunoblotted for caspases and caspase substrates.

To examine whether PARK2 activated the mitochondrial apoptosis pathway upon antimicrotubule drug treatment, immunofluorescence assays were performed. Docetaxel-treated MCF-7-PARK2 cells were stained with immunofluorescent BAX (6A7) antibody (this antibody specifically detects the activated form of BAX) 28. Overexpression of PARK2 significantly promoted BAX activation and translocation into the mitochondria in MCF-7 treated with docetaxel (Figure 4A). Meanwhile, fractionation assay of the mitochondria showed that overexpression of PARK2 resulted in significant increase in BAX oligomerization following docetaxel treatment of MCF-7 cells (Figure 4B). JC-1 assay showed that overexpression of PARK2 significantly reduced the mitochondrial membrane potential after docetaxel treatment of MCF-7 cells (Figure 4C). This is consistent with a previous report that activated BAX dimer translocated into the mitochondria promoting decrease of mitochondrial membrane potential (Δψm) 29. Moreover, we observed that overexpression of PARK2 in MCF-7 cells significantly promoted the mitochondrial release of cytochrome C (Figure 4D), followed by activated proteolytic caspases (Figure 4E). To examine whether endogenous high expression of PARK2, activated and translocated BAX to the mitochondria upon docetaxel treatment, immunofluorescence assay and western blot were performed using MDA-MB-134-VI cells. We found that BAX protein was activated and translocated into the mitochondria in MDA-MB-134-VI cells after docetaxel treatment. ShRNA silencing of endogenous PARK2 resulted in significantly decrease BAX oligomerization and mitochondrial translocation (Figure S3A&B).

In summary, PARK2 significantly activated the mitochondrial apoptotic pathway after docetaxel exposure.

### Antimicrotubule drugs promote the interaction between PARK2 and phospho-BCL-2

**Figure 5:**
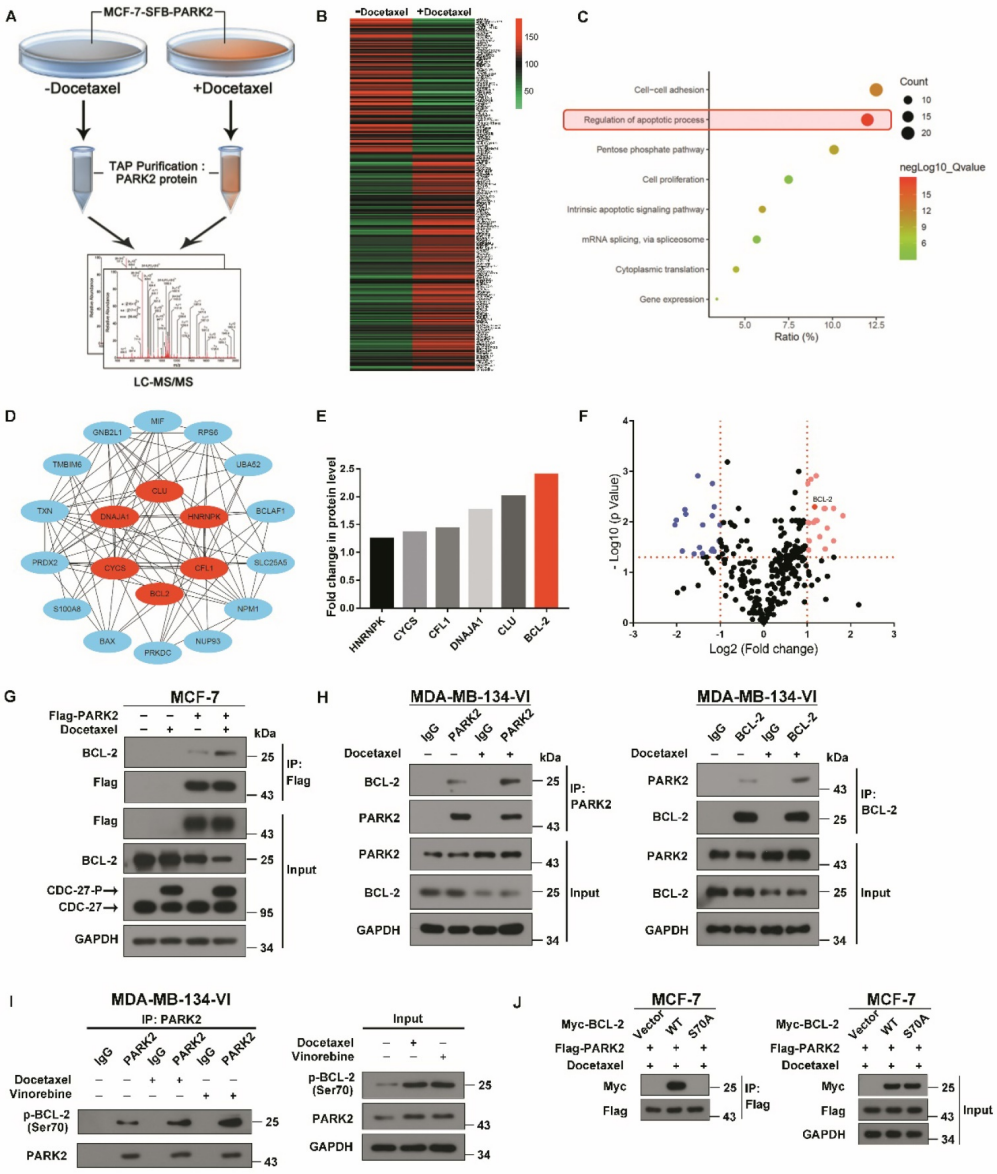
** Antimicrotubule drug promotes interaction between PARK2 and BCL-2. A.** Proteomics workflow diagram. (MCF-7-SFB-PARK2: MCF-2 stably expressing SFB tag PARK2). **B.** Heatmap cluster of proteomic changes with binding to PARK2 before and after docetaxel treatment in MCF-7-PARK2 cell line. **C.** Gene Ontology (GO) analysis of increased proteins after docetaxel treatment. **D.** PPIN analysis of apoptosis associated proteins to search hub proteins. (Red nodes represent hub proteins, blue nodes represent non-important proteins, and line represent the relationship between two proteins).** E.** Fold change in protein level of 6 selected hub proteins. Illustrated is the fold change in protein level of post-docetaxel treatment compared to pre-docetaxel treatment. **F.** Volcanic map of proteomic changes with binding to PARK2 either before or after docetaxel treatment. Vertical dashed lines indicate cut-off of log2Fold change (1 or -1). Horizontal dashed lines indicate the cut-off of p-value (0.05). **G.** MCF-7 cells stably expressing either ectopic wild-type PARK2 or vector (control) were treated with docetaxel (1 nM) for 24 h. Cells were lysed with RIPA buffer followed by immunoprecipitation (IP) using anti-FLAG agarose and western blot with indicated antibody. **H&I.** MDA-MB-134-VI cells were treated with either docetaxel or vinorelbine for 24 h. After IP assays were performed with endogenous PARK2, BCL-2 or phospho-BCL-2 (Ser70) in MDA-MB-134-VI cells. **J.** Association of Flag-PARK2 with Myc-BCL-2 mutants S70A.

To investigate the molecular mechanisms by which PARK2 activates the mitochondrial apoptosis pathway after exposure to antimicrotubule drugs, in-depth proteomic profiling was performed. Proteomic changes of protein binding to PARK2 before and after docetaxel treatments of MCF-7-PARK2 cells were analyzed (Figure 5A). In total, 290 differentials PARK2 binding proteins were identified (Table S1) with excellent reproducibility (Figure S4A). Docetaxel treatment of MCF-7-PARK2 cells exhibited a highly distinct enrichment of binding proteins compared to non-treated cells (Figure 5B). Based on the Gene Ontology (GO) Analysis, apoptosis-related proteins were significantly enriched after docetaxel treatment, with 20 proteins associated with apoptosis (Figure 5C). The hub protein is the one that plays a vital role in the biological processes. In related pathways, the regulation of other gene is often affected by the hub protein 30, 31. To determine which gene among the 20 genes is the hub protein, protein-protein interaction networks (PPIN) based on the STRING database were constructed through the cytoHubaa plug-in unit. We screened 6 hub proteins by PPIN analysis (Figure 5D). To narrow our list of candidate targets, we focused on 6 hub proteins that exhibited the most robust differences between the groups of pre- and post- docetaxel treatment. BCL-2 protein was the most highly enriched after docetaxel treatment (Figure 5E), with a statistically significant *p*-value (Figure 5F). Exogenous co-immunoprecipitation (co-IP) assays further confirmed that PARK2 interacted with BCL-2 before docetaxel treatment, while this interaction markedly increased after docetaxel treatment as determined on western blot (Figure 5G). Endogenous co-immunoprecipitation (co-IP) assays also supported this conclusion (Figure 5H). The interaction between PARK2 and BCL-2 also increased following vinorelbine treatment (Figures S4B and S3C).

Antimicrotubule drugs induce phosphorylation of BCL-2 on its serine (Ser) residues Ser70 (5). We examined whether PARK2 interacts with phospho-BCL-2. First, we examined antimicrotubule drugs versus DNA-damaging drugs. We found the former induced the phosphorylation of BCL-2 (Figure S4D) which is consistent with previous reports 26, 32, 33. We also examined whether phosphorylation of BCL-2 at ser70 enhances BCL-2 antiapoptotic functions in breast cancer cell. We abrogated BCL-2 phosphorylation by introducing a conserved, non-phosphorylated alanine (A) at ser70 sites. We mimic BCL-2 phosphorylation by introducing a glutamate (E) at the ser70 sites. Mutant BCL-2 was stably transfected of MCF-7 cells, and clones expressing quantitatively similar levels of BCL-2 were selected and tested (Figure S4E). Flow cytometry assay showed that BCL-2 mutant S70E significantly reduced apoptosis after docetaxel treatment in MCF-7 cells (Figure S4F). MTT assays showed that BCL-2 mutant S70E rendered cells more resistant to docetaxel in MCF-7 cells (Figure S4G). However, BCL-2 mutant S70A had much reduce antiapoptotic activity when compared with BCL-2-WT. These results demonstrated that phosphorylation of BCL-2 at ser70 enhanced BCL-2 antiapoptotic functions. Endogenous immunoprecipitation (IP) assays showed that PARK2 interacts with phospho-BCL-2 (Ser70) before docetaxel treatment, while this interaction markedly increased after docetaxel treatment (Figure 5I). Furthermore, BCL-2 mutants S70A did not interact with PARK2 (Figure 5J). Taken together, PARK2 interacted with phospho-BCL-2 (Ser70).

Collectively, these data indicated that antimicrotubule drugs promoted the interaction between PARK2 and phospho-BCL-2.

### PARK2 promotes poly-ubiquitination and degradation of BCL-2

**Figure 6:**
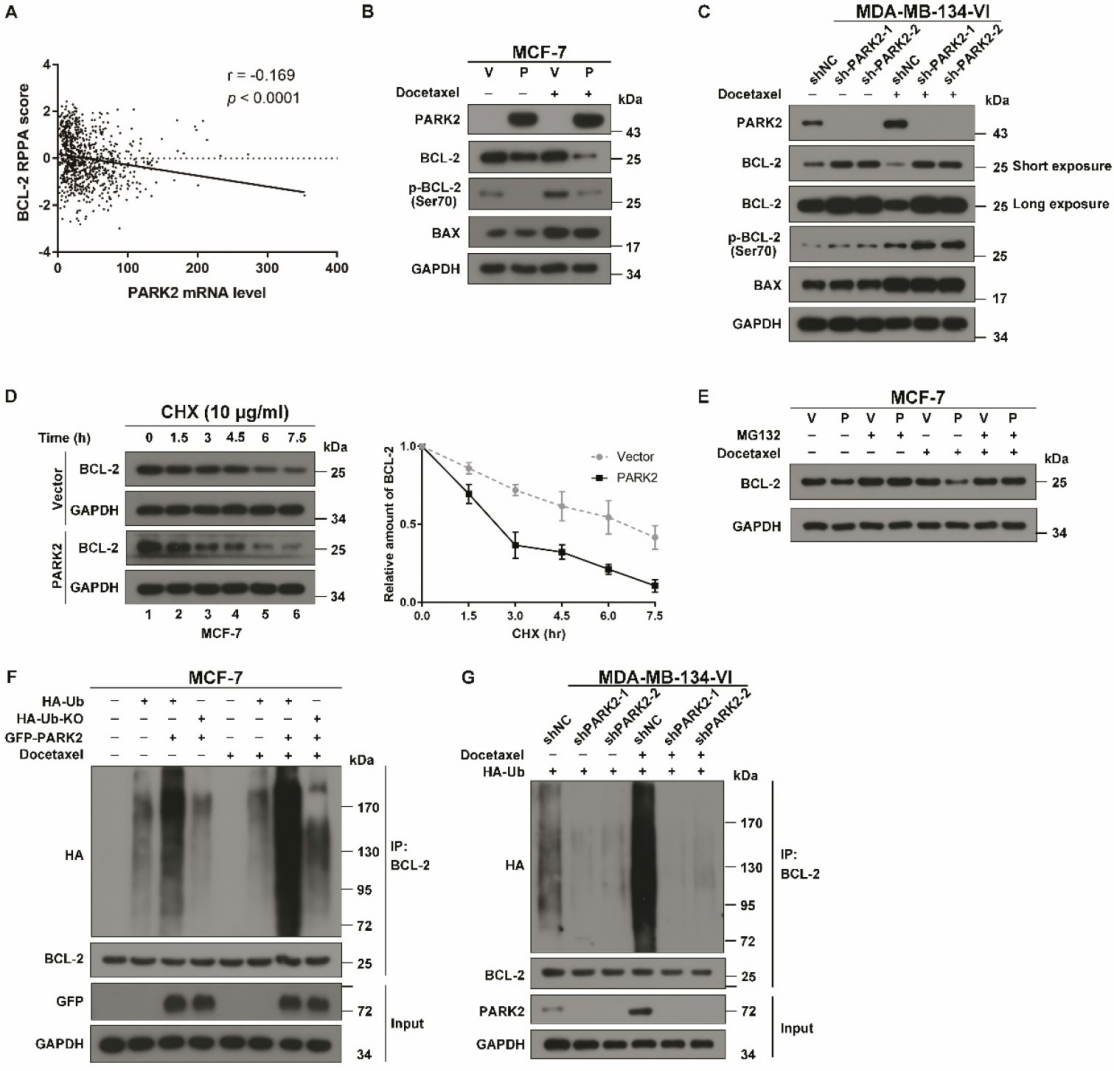
** PARK2 promotes poly-ubiquitination and degradation of BCL-2. A.** Boxplots of RPPA data showed a negative correlation between the mRNA levels of PARK2 and protein levels of BCL-2. B. MCF-7 cells stably expressing either ectopic wild-type PARK2 (P) or vector control (V) were treated with docetaxel (1 nM) for 24 h. C. MDA-MB-134-VI cells stably deleting either endogenous PARK2 or shNC control were treated with docetaxel (2 nM) for 24 h. **B-C.** Cell lysates were immunoblotted for BCL-2, phospho-BCL-2 (Ser70), BAX, PARK2, and GAPDH. **D.** MCF-7 cells stably expressing either ectopic wild-type PARK2 or vector (control) were treated with docetaxel (1 nM) for 24 h and then incubated with 10 µg/ml cycloheximide (CHX) for the indicated periods of time and then immunoblotted (Left panel). Quantification of BCL-2 protein levels, n = 3. Error bars indicate SEM. Relative level of BCL-2 protein is normalized to 0 h (Right panel). **E.** MCF-7 cells stably expressing either ectopic wild-type PARK2 or vector control were treated either with or without docetaxel (1 nM) for 24 h. These cells were then cultured in the presence of either DMSO control or MG132 (10 mM) (6 h) followed by western blot analysis of BCL-2 levels. **F.** MCF-7 cells were transfected with indicated vectors for 18 h. Cells were then treated with or without docetaxel (1 nM) for 24 h. After docetaxel treatment, Cells were treated with MG132 (10 mM) and subjected to immunoprecipitation (IP) analysis with BCL-2 antibody. HA-Ub KO mutant (incapable of forming polyubiquitin chain) was used as a negative control. **G.** MDA-MB-134-VI cells stably expressing indicated shRNAs were transfected with indicated vectors for 24 hs. Cells were then treated with MG132 (10 mM) either in the presence or absence of docetaxel (1 nM) for 36 h and subjected to immunoprecipitation (IP) analysis with BCL-2 antibody.

We have experimentally demonstrated that antimicrotubule drugs promote the interaction between PARK2 and BCL-2; however, what is the relationship between PARK2 and BCL-2? An analysis of the TCGA dataset showed that BCL-2 protein levels were negatively correlated with PARK2 mRNA expression in breast cancers (Figure 6A). A total of 70% of breast cancers had a negative correlation between levels of PARK2 protein and BCL-2 protein according to The Human Protein Atlas database (Figure S5A & B). As PARK2 has E3 ligase activity, we hypothesized that PARK2 might promote BCL-2 degradation. To confirm this hypothesis, a series of western blotting assays were performed, showing that BCL-2 was slightly downregulated in MCF-7-PARK2 cell; however, BCL-2 was significantly decreased after docetaxel treatment of MCF-7-PARK2 cells (Figure 6B). Meanwhile, PARK2 overexpression markedly reduced levels of phospho-BCL-2 (Ser70) (Figure 6B). Silencing of endogenous PARK2 slightly elevated BCL-2 protein, but BCL-2 was dramatically increased after docetaxel treatment in sh-PARK2-MDA-MB-134-VI cells (Figure 6C). Silencing of endogenous PARK2 also significantly increased levels of phospho-BCL-2 (Ser70) (Figure 6C). Docetaxel treatment resulted in a substantial accumulation of the pro-apoptotic protein BAX; however, PARK2 expression had no effect on BAX levels (Figures 6B & C). Cycloheximide chase assay showed that after docetaxel treatment, cells overexpressing PARK2 caused a dramatic reduction in the half-life of BCL-2 (Figure 6D). Furthermore, proteasome inhibitor MG132 mitigated downregulation of BCL-2 confirming that PARK2-mediated degradation of antiapoptotic protein BCL-2 was proteasome-dependent (Figure 6E). Overexpression of PARK2 increased the level of ubiquitination of BCL-2; and this was more evident after docetaxel treatment (Figure 6F). Consistently, regardless of docetaxel treatment, silencing of endogenous PARK2 reduced ubiquitination of BCL-2 (Figure 6G). In addition, overexpression of PARK2 significantly downregulated BCL-2 protein levels in MCF-7 cells in response to vinorelbine treatment (Figure S5C). Silencing of PARK2 markedly increased BCL-2 protein levels in MDA-MB-134-VI cells after vinorelbine treatment (Figure S5D).

Moreover, immunofluorescence assay showed that phospho-BCL-2 (Ser70) was recruited to the mitochondria in MCF-7 cells after docetaxel treatment, whereas overexpression of PARK2 significantly reduced the recruitment of phospho-BCL-2 (Ser70). Meanwhile, overexpression of PARK2 markedly reduced fluorescence intensity of BCL-2 and phospho-BCL-2 (Ser70) after docetaxel treatment (Figure S5E).

Together, these data identified that PARK2 promoted degradation of the antiapoptotic protein BCL-2 through the ubiquitin-proteasome pathway, and this was markedly enhanced by antimicrotubule drugs.

### Following antimicrotubule drugs treatment, PARK2 is not able to induce cell death when BCL-2 is not degraded

Degradation of BCL-2 was blocked by MG132 in the presence of docetaxel treatment condition and then checked for activation of BAX. Immunofluorescence assay showed that overexpression of PARK2 was not able to promote BAX activation and translocation into the mitochondria in MCF-7 cells after MG132 treatment (Figure S6A). Meanwhile, fractionation assay of the mitochondrial showed that overexpression of PARK2 was not able to promote BAX oligomerization in MCF-7 cells after MG132 treatment (Figure S6B). Further, we observed that with MG132 treatment, overexpression of PARK2 was not able to promote the mitochondrial release of cytochrome C (Figure S6C). Overexpression of PARK2 was not able to activate proteolytic caspases following MG132 treatment of MCF-7 cells (Figure S6D). Overexpression of PARK2 is not able to induce cell apoptosis after mg132 treatment (Figure S6E).

In summary, PARK2 was not able to activate BAX and thus was not able to induce cell death when BCL-2 was not degraded.

### Under the condition of antimicrotubule drugs treatment, the pro-apoptotic effect of PARK2 is link to BCL-2, not to BCL-XL and MCL-1

**Figure 7:**
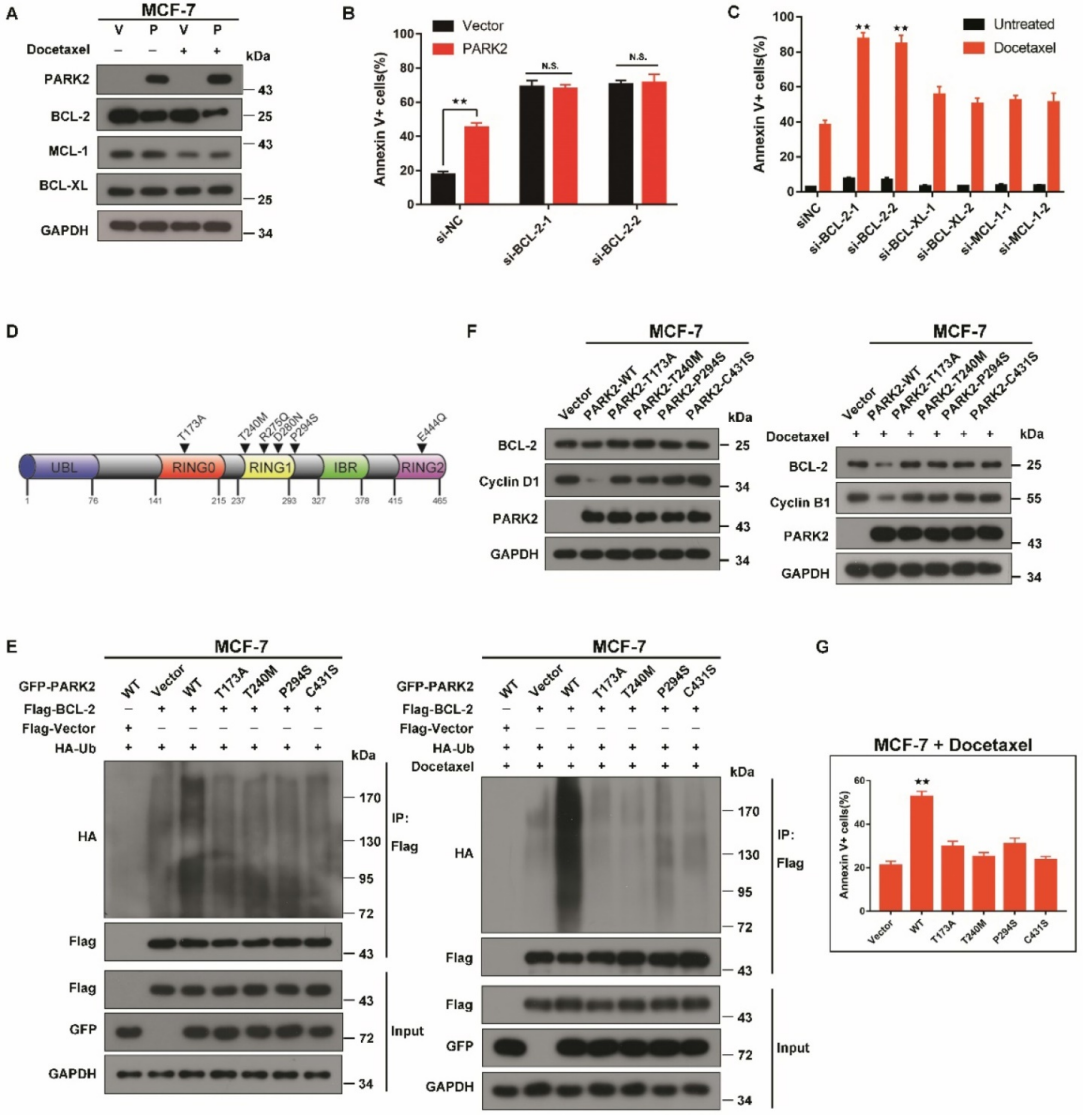
** PARK2 loss-of-function mutants fail to enhance antimicrotubule drugs sensitivity A.** MCF-7 cells stably expressing either ectopic wild-type PARK2 (P) or vector control (V) were treated with docetaxel (1 nM) for 24 h. **B.** MCF-7 cells stably expressing either ectopic wild-type PARK2 (P) or vector control (V) were transfected with control siRNA or siRNA targeted against BCL-2 for 56 h. After transfection for 24 h, cells were treated with docetaxel for 48h. **C.** MCF-7-PARK2 cells were transfected with control siRNA or siRNA targeted against the indicated BCL-2 family member for 50 h. Knockdown efficiency was analyzed by western blotting (Supplementary Figure. S5A). Cells were treated with docetaxel for 48h. **D.** Pan-cancer analysis of PARK2 loss-of-function mutations. **E.** MCF-7 cells (Vector (control), PARK2 wild type (WT), T173, T240M, P294S, and C431S clones) were transfected with indicated plasmid for 18 h. Cells were then treated either without (left panel) or with (right panel) docetaxel (1 nM) for 24 h. MG132 was added 6 h prior to harvest cell lysate. Cells were lysed with RIPA buffer followed by immunoprecipitation (IP) using anti-FLAG agarose and western blot was done with indicated antibody. **F.** Overexpression of wild-type but not loss-of-function mutants of PARK2 led to lower levels of BCL-2 protein. Cyclin D1 and Cyclin B1 protein was used as a positive control. **G.** Overexpression of wild-type but not loss-of-function PARK2 mutants increased apoptosis in breast cancer cells after docetaxel (1 nM) treatment. Data show mean ± s.d. N = 3. **P < 0.01.

Because PARK2 significantly promoted BAX activation and activated the mitochondrial pathway of apoptosis after exposure to antimicrotubule drugs, we considered the possibility that PARK2 might promote degradation of more BCL-2 family proteins to promote BAX activation, leading to apoptosis. Therefore, we monitored the stability of the major antiapoptotic BCL-2 family proteins expressed in MCF-7 cells (BCL-2, BCL-XL and MCL-1) in response to antimicrotubule drugs. BCL-2, but not BCL-XL, MCL-1 was significantly downregulated after docetaxel treatment of MCF-7-PARK2 cells (Figure 7A). PARK2 mediated sensitivity to docetaxel was blocked through knockdown of BCL-2, suggesting that PARK2 mediated-antimicrotubule drugs sensitivity is mediated by downregulation of BCL-2 (Figure 7B and S7A). Because the preceding results suggested that PARK2 promoted degradation of BCL-2 after exposure to antimicrotubule drugs, we wondered whether knockdown of BCL-2 was sufficient to sensitize antimicrotubule-induced apoptosis. As controls, we also silenced other members of the antiapoptotic subset of the BCL-2 family (BCL-XL and MCL-1). Knockdown of BCL-2 in MCF-7-PARK2 cells greatly sensitized apoptosis induced by antimicrotubule drugs. In contrast, knockdown of BCL-XL and MCL-1 had much more modest effects (Figure 7C and S7A).

### PARK2 enhancing antimicrotubule drugs sensitivity depends on its E3 ubiquitin ligase activity

PARK2 point mutations can abolish E3 ligase activity 34. Interestingly, in the cBioPortal database, PARK2 loss-of-function mutants (T173A, T240M, R275Q, D280N, P294S, and E444Q) were found in several human cancers (Figure 7D). As the degradation of BCL-2 by PARK2 is dependent on its E3 ubiquitin ligase activity, we speculated that PARK2 loss-of-function mutants would not promote BCL-2 degradation. We chose three tumor-associated PARK2 loss-of-function mutations (T173A, T240M, P294S), as well as a known ligase-dead mutant C431S. Ubiquitination assays were performed on MCF-7 cells. Compared with wild-type PARK2, all of the loss-of-function mutants showed decreased ubiquitination of BCL-2 (Figure 7E and S7B). These loss-of-function mutations blocked the degradation of BCL-2 (Figure 7F and S7C). Furthermore, these mutations failed to enhance antimicrotubule drugs sensitivity (Figure 7G and S7D).

These data demonstrated that the PARK2 loss-of-function mutations blocked the degradation of BCL-2 and failed to increase antimicrotubule drugs sensitivity.

### PARK2 enhances antimicrotubule drug sensitivity *in vivo*

**Figure 8:**
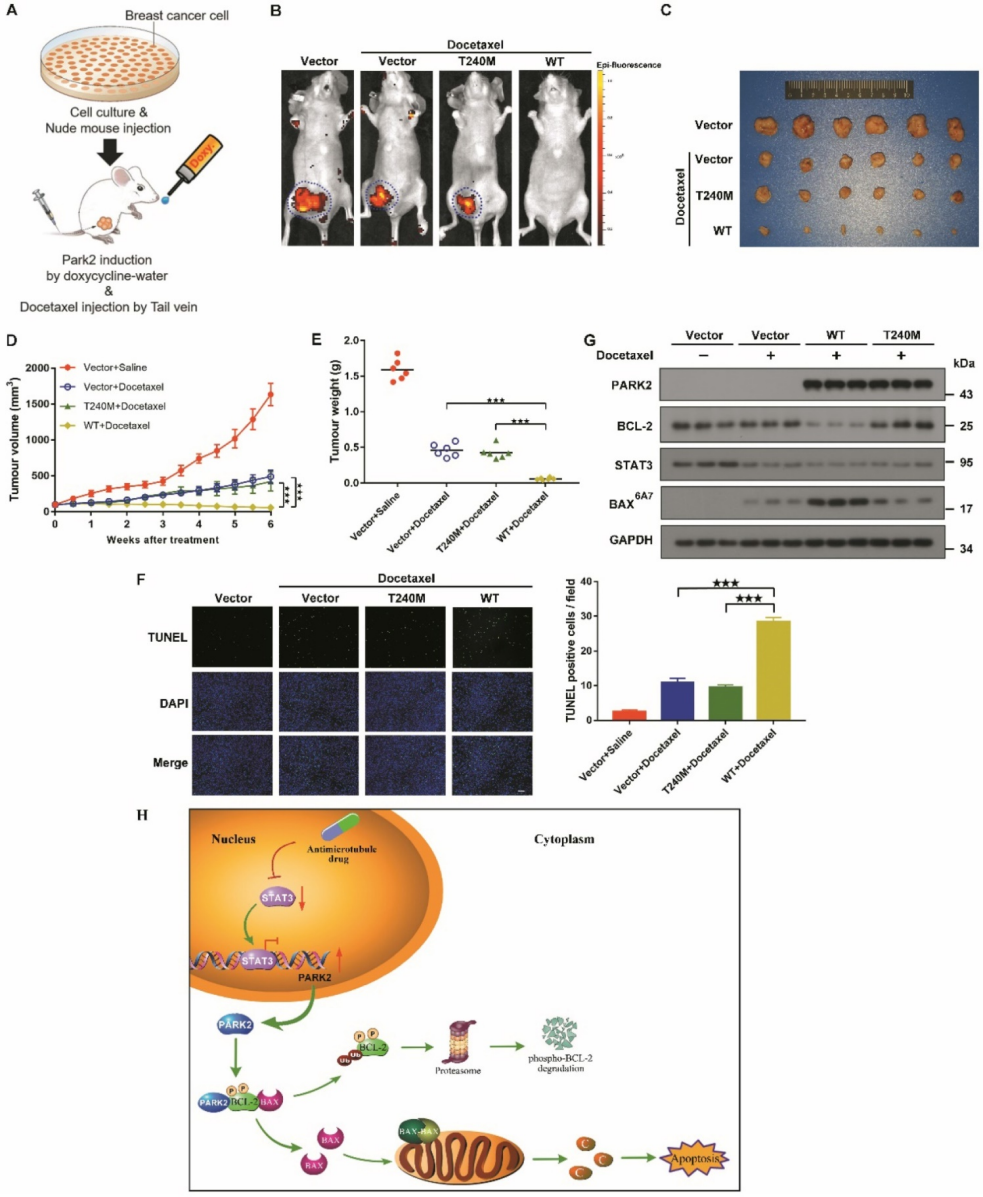
** PARK2 enhances antimicrotubule drug sensitivity *in vivo*. A.** Experimental schematic. G. BALB/c nude mice were injected with MCF-7 cells which were stably transfected with doxycycline-inducible, fluorescently-labeled constructs (Vector, PARK2-WT and PARK2-T240M) in the breast orthotopic model. When tumors reached 100 mm3, doxycycline was added in the mice drinking water, and docetaxel (10 mg/kg) was injected through tail vein at the same time. **B.** Fluorescence images of tumor in BALB/c nude mice injected with fluorescently-labeled MCF-7 cells overexpressing vector (control), wild-type PARK2 or PARK2-T240M. Fluorescence intensity of wild-type PARK2 tumors were too low to be detected. **C.** Images of resected tumors at the end point (day 42). **D-E.** When tumors reached 100 mm3, tumor volumes at the indicated times were measured. Weights of resected tumors at the end point (day 42) (n=6). **F.** Representative immunofluorescent images for apoptotic cells were examined by TUNEL staining in tumor tissues. Scale bar represents 20 µm. And quantification of apoptotic cells for each group (n = 6). **G.** Expression of BCL-2, BAX (6A7), STAT3 and PARK2 were detected by Western blot in protein lysates of tumor tissue. **H.** Model illustrates that following antimicrotubule drugs treatment, the expression of PARK2 was upregulated due to the repression of STAT3-mediated transcriptional inhibition of PARK2. PARK2 promotes degradation of phospho-BCL-2 following antimicrotubule drugs treatment, which activates the mitochondrial apoptotic pathway. Data show mean ± s.d. N = 6, ***P < 0.001.

To confirm further whether PARK2 contributes to antimicrotubule drugs sensitivity* in vivo*, we established a breast cancer xenograft model (Figure 8A). Doxycycline-inducible MCF-7 cells were also fluorescently-labeled and overexpressed either for vector (control), wild-type PARK2 or loss-of-function mutant of PARK2 (PARK2-T240M). These cells were orthotopically injected. Induction of wild-type PARK2 expression significantly enhanced docetaxel sensitivity (Figures 8B-E). Furthermore, wild-type PARK2 dramatically increased apoptosis of the tumor cells (Figure 8F). In contrast, PARK2-T240M had no effect on docetaxel sensitivity *in vivo* (Figures 8B-F). Docetaxel decrease STAT3 level. Wild-type PARK2, but not PARK2-T240M, markedly reduced BCL-2 levels and increased BAX activation following docetaxel treatment (Figure 8G).

Our data showed that PARK2 enhanced antimicrotubule drug sensitivity by promoting the degradation of BCL-2* in vivo.*

## Discussion

Increasing efforts to identify what chemotherapy will be effective for individual patients underscore the need for applicable biomarkers to predict response to specific drug, in order to achieve effective personalized treatment while saving patients from side effects. Here, for the first time, we demonstrated that PARK2 regulates sensitivity to antimicrotubule drugs (Figure 8H) and provide a potential biomarker for predicting response to antimicrotubule drugs in breast cancer.

In our present study, we analyzed expression of PARK2 in 205 tumor samples from breast cancer patients. We found that for patients who received antimicrotubule drugs, highly expressed PARK2 in breast cancer tissues was strongly associated with higher rate of pCR (Figure 1H). Meanwhile, higher expression of PARK2 also showed better DFS and overall survival (Figure 1F). In contrast, for the patients who received DNA-damaging drugs (without antimicrotubule drugs), no significant differences were observed in their DFS and overall survival, regardless of the expression of PARK2 in the tumors (Figure 1F). *In vivo* and *in vitro*, PARK2 sensitized apoptosis induced by antimicrotubule drugs (e.g. docetaxel and vinorelbine) in breast cancer cells (Figure 2A). This effect was highly selective and was not observed in DNA-damaging drugs (e.g. adriamycin, cyclophosphamide and 5-fluorouracil) (Figure S2B). Thus, efficacy of antimicrotubule drugs, but not DNA-damaging drugs, is associated with levels of PARK2.

We investigate the molecular mechanisms by which PARK2 mediated-antimicrotubule drugs sensitivity. We found that PARK2 significantly activated the mitochondrial apoptotic pathway after docetaxel treatment (Figure 4A-E). Meanwhile, our in-depth proteomic profiling showed that BCL-2 protein was the most highly enriched protein binding to PARK2 after docetaxel treatment. A series of co-immunoprecipitation (co-IP) assays also confirmed that PARK2 interacted with BCL-2.

Antimicrotubule drugs induce cytoprotective stress responses that suppress apoptosis and promote chemoresistance. Antiapoptotic protein, BCL-2, plays a key role in these cytoprotective stress responses. BCL-2 is a crucial regulator of mitochondrial apoptotic pathway. Antimicrotubule drugs induced BCL-2 phosphorylation at Ser70 (5). BCL-2 phosphorylation greatly enhances antiapoptotic function of the molecular. Phospho-BCL-2 confers chemoresistance of antimicrotubule drugs 10, 12, 35. Furthermore, we found that PARK2 specifically interacts with phospho-BCL-2 (Ser70) which is augmented by antimicrotubule drugs. PARK2 degraded phospho-BCL-2 in an E3 ligase dependent manner (Figure 6A-G). Antimicrotubule drugs induced BCL-2 phosphorylation, whereas DNA-damaging drugs do not 26, 32, 33. This may explain why PARK2 selectively confer chemosensitivity to antimicrotubule drugs, but not to DNA-damaging drugs. Collectively, PARK2 significantly improves sensitivity to antimicrotubule drugs via degradation phospho-BCL-2.

Antiapoptotic family proteins include BCL-2 and its close relatives BCL-XL and MCL-1. PARK2 has also been reported to enhance apoptosis induced by mitochondrial depolarization drugs by promoting poly-ubiquitination and degradation of MCL-1 20. We considered the possibility that PARK2 might promote degradation of additional BCL-2 family proteins after antimicrotubule drugs treatment. We showed that BCL-2, but not BCL-XL, MCL-1 was significantly downregulated after docetaxel treatment of MCF-7-PARK2 cells (Figure 7A). Sensitivity to docetaxel was enhanced by PARK2 which was blocked by knockdown of BCL-2 (Figure 7B). Thus, under antimicrotubule drugs treatment, the pro-apoptosis effect of PARK2 is linked to BCL-2, not to BCL-XL and MCL-1.

In conclusion, we showed that elevated expression of PARK2 may play an important in enhancing sensitivity to antimicrotubule drugs in breast cancer. In addition, PARK2 mediated-antimicrotubule drug sensitivity is, at least in part, mediated by downregulation of phospho-BCL-2 which activated the mitochondrial pathway of apoptosis. Our studies provide a rationale for using PARK2 as a potential biomarker to predict chemosensitivity of antimicrotubule drugs-containing regimen.

## Supplementary Material

Supplementary figures and tables.

## Abbreviations

DFS: disease-free survival
pCR: pathologic complete response
ChIP: chromatin immunoprecipitation
Co-IP: coimmunoprecipitation
TF: transcription factors
Δψm: mitochondrial membrane potential
PPIN: protein-protein interaction networks

## References

[1] Iwata H, Sato N, Masuda N, Nakamura S, Yamamoto N, Kuroi K (2011). Docetaxel followed by fluorouracil/epirubicin/cyclophosphamide as neoadjuvant chemotherapy for patients with primary breast cancer. Jpn J Clin Oncol.

[2] Dieras V, Fumoleau P, Romieu G, Tubiana-Hulin M, Namer M, Mauriac L (2004). Randomized parallel study of doxorubicin plus paclitaxel and doxorubicin plus cyclophosphamide as neoadjuvant treatment of patients with breast cancer. J Clin Oncol.

[3] Nowak AK, Wilcken NR, Stockler MR, Hamilton A, Ghersi D (2004). Systematic review of taxane-containing versus non-taxane-containing regimens for adjuvant and neoadjuvant treatment of early breast cancer. Lancet Oncol.

[4] Jordan MA, Wilson L (2004). Microtubules as a target for anticancer drugs. Nature Reviews Cancer.

[5] Bhalla KN (2003). Microtubule-targeted anticancer agents and apoptosis. Oncogene.

[6] Zhai Y, Kronebusch PJ, Simon PM, Borisy GG (1996). Microtubule dynamics at the G2/M transition: abrupt breakdown of cytoplasmic microtubules at nuclear envelope breakdown and implications for spindle morphogenesis. J Cell Biol.

[7] Radha G, Raghavan SC (2017). BCL2: A promising cancer therapeutic target. Biochim Biophys Acta Rev Cancer.

[8] Yu Q, Qiu Y, Chen X, Wang X, Mei L, Wu H (2019). Chemotherapy priming of the Pancreatic Tumor Microenvironment Promotes Delivery and Anti-Metastasis Efficacy of Intravenous Low-Molecular-Weight Heparin-Coated Lipid-siRNA Complex. Theranostics.

[9] Li Y, Zhang B, Xiang L, Xia S, Kucuk O, Deng X (2020). TGF-beta causes Docetaxel resistance in Prostate Cancer via the induction of Bcl-2 by acetylated KLF5 and Protein Stabilization. Theranostics.

[10] Deng X, Gao F, Flagg T, May WS Jr (2004). Mono- and multisite phosphorylation enhances Bcl2's antiapoptotic function and inhibition of cell cycle entry functions. Proc Natl Acad Sci U S A.

[11] Deng X, Xiao L, Lang W, Gao F, Ruvolo P, May WS Jr (2001). Novel role for JNK as a stress-activated Bcl2 kinase. J Biol Chem.

[12] Ito T, Deng X, Carr B, May WS (1997). Bcl-2 phosphorylation required for anti-apoptosis function. J Biol Chem.

[13] Kitada T, Asakawa S, Hattori N, Matsumine H, Yamamura Y, Minoshima S (1998). Mutations in the parkin gene cause autosomal recessive juvenile parkinsonism. Nature.

[14] Park MH, Lee HJ, Lee HL, Son DJ, Ju JH, Hyun BK (2017). Parkin Knockout Inhibits Neuronal Development via Regulation of Proteasomal Degradation of p21. Theranostics.

[15] Beroukhim R, Mermel CH, Porter D, Wei G, Raychaudhuri S, Donovan J (2010). The landscape of somatic copy-number alteration across human cancers. Nature.

[16] Veeriah S, Taylor BS, Meng S, Fang F, Yilmaz E, Vivanco I (2010). Somatic mutations of the Parkinson's disease-associated gene PARK2 in glioblastoma and other human malignancies. Nat Genet.

[17] Gong Y, Zack TI, Morris LG, Lin K, Hukkelhoven E, Raheja R (2014). Pan-cancer genetic analysis identifies PARK2 as a master regulator of G1/S cyclins. Nat Genet.

[18] Lee SB, Kim JJ, Nam HJ, Gao B, Yin P, Qin B (2015). Parkin Regulates Mitosis and Genomic Stability through Cdc20/Cdh1. Mol Cell.

[19] Dawson TM, Dawson VL (2010). The role of parkin in familial and sporadic Parkinson's disease. Mov Disord.

[20] Carroll RG, Hollville E, Martin SJ (2014). Parkin sensitizes toward apoptosis induced by mitochondrial depolarization through promoting degradation of Mcl-1. Cell Rep.

[21] Hong J, Hu K, Yuan Y, Sang Y, Bu Q, Chen G (2012). CHK1 targets spleen tyrosine kinase (L) for proteolysis in hepatocellular carcinoma. J Clin Invest.

[22] Yuan J, Jiang YY, Mayakonda A, Huang M, Ding LW, Lin H (2017). Super-Enhancers Promote Transcriptional Dysregulation in Nasopharyngeal Carcinoma. Cancer Res.

[23] Walker SR, Chaudhury M, Nelson EA, Frank DA (2010). Microtubule-targeted chemotherapeutic agents inhibit signal transducer and activator of transcription 3 (STAT3) signaling. Mol Pharmacol.

[24] Zhang L, Xu X, Yang R, Chen J, Wang S, Yang J (2015). Paclitaxel attenuates renal interstitial fibroblast activation and interstitial fibrosis by inhibiting STAT3 signaling. Drug Des Devel Ther.

[25] Zhang X, Wu X, Zhang F, Mo S, Lu Y, Wei W (2017). Paclitaxel induces apoptosis of esophageal squamous cell carcinoma cells by downregulating STAT3 phosphorylation at Ser727. Oncol Rep.

[26] Wang LG, Liu XM, Kreis W, Budman DR (1999). The effect of antimicrotubule agents on signal transduction pathways of apoptosis: a review. Cancer Chemother Pharmacol.

[27] Rosse T, Olivier R, Monney L, Rager M, Conus S, Fellay I (1998). Bcl-2 prolongs cell survival after Bax-induced release of cytochrome c. Nature.

[28] Hsu YT, Youle RJ (1997). Nonionic detergents induce dimerization among members of the Bcl-2 family. J Biol Chem.

[29] Tait SW, Green DR (2010). Mitochondria and cell death: outer membrane permeabilization and beyond. Nat Rev Mol Cell Biol.

[30] Zhou Z, Cheng Y, Jiang Y, Liu S, Zhang M, Liu J (2018). Ten hub genes associated with progression and prognosis of pancreatic carcinoma identified by co-expression analysis. Int J Biol Sci.

[31] Chin CH, Chen SH, Wu HH, Ho CW, Ko MT, Lin CY (2014). cytoHubba: identifying hub objects and sub-networks from complex interactome. BMC Syst Biol.

[32] Blagosklonny MV, Giannakakou P, el-Deiry WS, Kingston DG, Higgs PI, Neckers L (1997). Raf-1/bcl-2 phosphorylation: a step from microtubule damage to cell death. Cancer Res.

[33] Haldar S, Basu A, Croce CM (1997). Bcl2 is the guardian of microtubule integrity. Cancer Res.

[34] Trempe JF, Sauve V, Grenier K, Seirafi M, Tang MY, Menade M (2013). Structure of parkin reveals mechanisms for ubiquitin ligase activation. Science.

[35] Wei Y, Pattingre S, Sinha S, Bassik M, Levine B (2008). JNK1-mediated phosphorylation of Bcl-2 regulates starvation-induced autophagy. Mol Cell.

