# Supplementary material for: PARK2 promotes mitochondrial pathway of apoptosis and antimicrotubule drugs chemosensitivity via degradation of phospho-BCL-2
Source: Theranostics. 2020 Aug 8;10(22):9984–10000. doi: 10.7150/thno.47044 (PMC7481404; doi:10.7150/thno.47044)
Supplement: Supplementary file 1 — Supplementary figures and tables. [file thnov10p9984s1.pdf]

Supplementary Information

PARK2 promotes mitochondrial pathway of apoptosis and antimicrotubule drugs chemosensitivity via degradation of phospho-BCL-2

Hengxing Chen[1 †], Yun Li[1 †], Yu Li[1], Zhen Chen[1], Limin Xie[1], Wenjia Li[1], Yuanxin Zhu[1], Hong Xue[2], H. Phillip Koeffler[3,4], Wenjing Wu[5], Kaishun Hu[1 *], Dong Yin[1, *]

Table of contents

Supplementary Materials and Methods……………………………………………2

Supplementary Figure. 1……………………………………………………………6

Supplementary Figure. 2……………………………………………………………7

Supplementary Figure. 3……………………………………………………………8

Supplementary Figure. 4……………………………………………………………9

Supplementary Figure. 5……………………………………………………………10

Supplementary Figure. 6……………………………………………………………11

Supplementary Figure. 7……………………………………………………………12

Supplementary Table 1………………………………………..………………13

Supplementary Table 2…………………………………………………..………22

Supplementary Materials and Methods

Cell lines

The human breast cell lines MCF-7, MDA-MB-231, T47D, ZR-75-30 and MDA-MB-134-VI were obtained from ATCC. MDA-MB-231 were maintained in DMEM supplemented with 10% (v/v) FBS and 1% (v/v) Penicillin-Streptomycin, at 37°C, 5% CO2 in a humidified atmosphere. MCF-7, T47D and ZR-75-30 grew in RPMI 1640 with 10% fetal bovine serum and 1% Penicillin-Streptomycin. MDA-MB-134-VI were cultured in Leibovitz's L-15 Medium with 20% (v/v) FBS and 1% (v/v) Penicillin-Streptomycin.

Flow cytometric analysis

Cells were treated with designated chemotherapy drugs and then collected for Annexin V and JC-1 analyses. For Annexin V analysis, apoptosis in breast cancer cells was detected using the Annexin V-FITC Apoptosis Detection Kit (Invitrogen) according to the manufacturer's protocol. In brief, cells were incubated with 200µL of binding buffer containing 5µL of FITC-conjugated Annexin V antibody for 10min at room temperature in the dark. After incubation, samples were resuspended in binding buffer (200µL) containing 10µL of Propidium Iodide Staining Solution and analyzed by flow cytometry.

For JC-1 analyses, MCF-7 cells were treated with docetaxel for 24 h and then collected. The mitochondria membrane potential in MCF-7 cells was detected using the JC-1 Assay Kit (Invitrogen). Briefly, cells were incubated with 500 µL of PBS containing 2mol JC-1 for 10

min at 37°C. After incubation, cells were centrifugated and the supernatant was removed. Samples were washed with PBS one time. The samples were then analyzed by flow cytometry.

Chromatin immunoprecipitation (ChIP) assay

To cross-link DNA and protein, 2 X 10^7 of ZR-75-30 and MDA-MB-134-VI cells were fixed with 1% formaldehyde for 10 min at room temperature. For immunoprecipitation, solubilized chromatin was incubated with 5ug of anti-STAT3 antibody, or IgG control overnight at 4°C on a rotating wheel. Antibody-chromatin complexes were subsequently pulled down by incubating with Dyna beads Protein G (Life Technologies) for 4 h at 4°C. After reversal of crosslink, RNase A as well as Proteinase K treatment, immunoprecipitated DNA was extracted with the Min-Elute PCR purification kit (Qiagen), followed by qPCR analysis. Primers for qPCR analysis are listed in Supplementary Table 2.

Luciferase reporter assay

PARK2 promoter (~500bp) was cloned into the Firefly luciferase reporter vector pGL3-Promoter (Promega). 1 X 10^5 of MDA-MB-134-VI cells were seeded into 24-well plates for 24 hours. Then, cells were transfected with si-NC, si-STAT3, pGL3-Control vector, pGL3-PARK2-promoter vector using Lipofectamine 2000 transfection reagent. After 24 hours of transfection, cells were collected, and the assay was performed following the manufacturer's instructions. Luciferase activity was measured using the Dual-Luciferase Reporter Assay System (Promega).

Fluorescence microscopy

MCF-7 cells were plated onto glass coverslips and treated with docetaxel for 24 h. MCF-7 cells were fixed with paraformaldehyde for 20 min and stained using standard protocols. Immunofluorescence images were detected with a ZEISS confocal microscope (ZEISS-800, Germany) equipped with ZENblue2.3 software.

Tumor xenografts

Three million MCF-7 cells, stably transduced with doxycycline-inducible and fluorescence-labeled constructs (Vector, PARK2 WT and PARK2-T240M) were mixed at a 1:1 dilution of Matrigel solution (BD Biosciences). Meanwhile, Estrogen piece were subcutaneous implanted in the of BALB/c nude mice. The mice were divided into four groups, two of which were vector groups, one was PARK2 WT, and the other was PARK2 T240M. Each group had six mice. The vector groups were treated with saline or docetaxel, respectively. The PARK2 WT group and PARK2 T240M group were treated with docetaxel. When tumors reached 100 mm3, doxycycline was administered in drinking water and mice were injected with docetaxel (10 mg/kg) one time per week. Tumor volume was measured every 3 days for 6 weeks and calculated using the following formula: volume (mm3) = [width (mm)] 2 X length(mm)/2.

TUNEL Assay

TUNEL assay was performed by using the In Situ Cell Death Detection Kit, POD at 37°Cfor 30 min. The slides were incubated with Alexa Fluor-conjugated secondary antibodies

(Invitrogen) for 1hr at room temperature. For DAPI staining, slides were incubated for 1hr at room temperature with a mounting medium for fluorescence containing DAPI. Images were obtained with a laser scanning confocal microscope (LSM780, Zeiss).

Isolation of mitochondrial and cytoplasmic fractions

Isolation of subcellular fractionation was performed using the Mitochondria Isolation Kit for Cultured Cells (Thermo Fisher) according to the manufacturer's protocol. Briefly, 10^8 MCF-7 cells were collected and added to 800μL of Mitochondria Isolation Reagent A. Vortex was set to medium and tubes were incubated on ice. 10μL of Mitochondria Isolation Reagent B was added to the tube and incubated for 5 min on ice. 800μL of Mitochondria Isolation Reagent C was added to the tube and then centrifuged at 700 X g for 10 min at 4°C. The supernatant was transferred to a new tube and centrifuged at 12,000 x g for 15 min at 4°C. The supernatant was cytosol fraction. 500μL of Mitochondria Isolation Reagent C was added to the pellet, centrifuged at 12,000 x g for 5 min and then the supernatant was discarded. Mitochondria was lysed with 2% CHAPS in Tris-buffered saline.

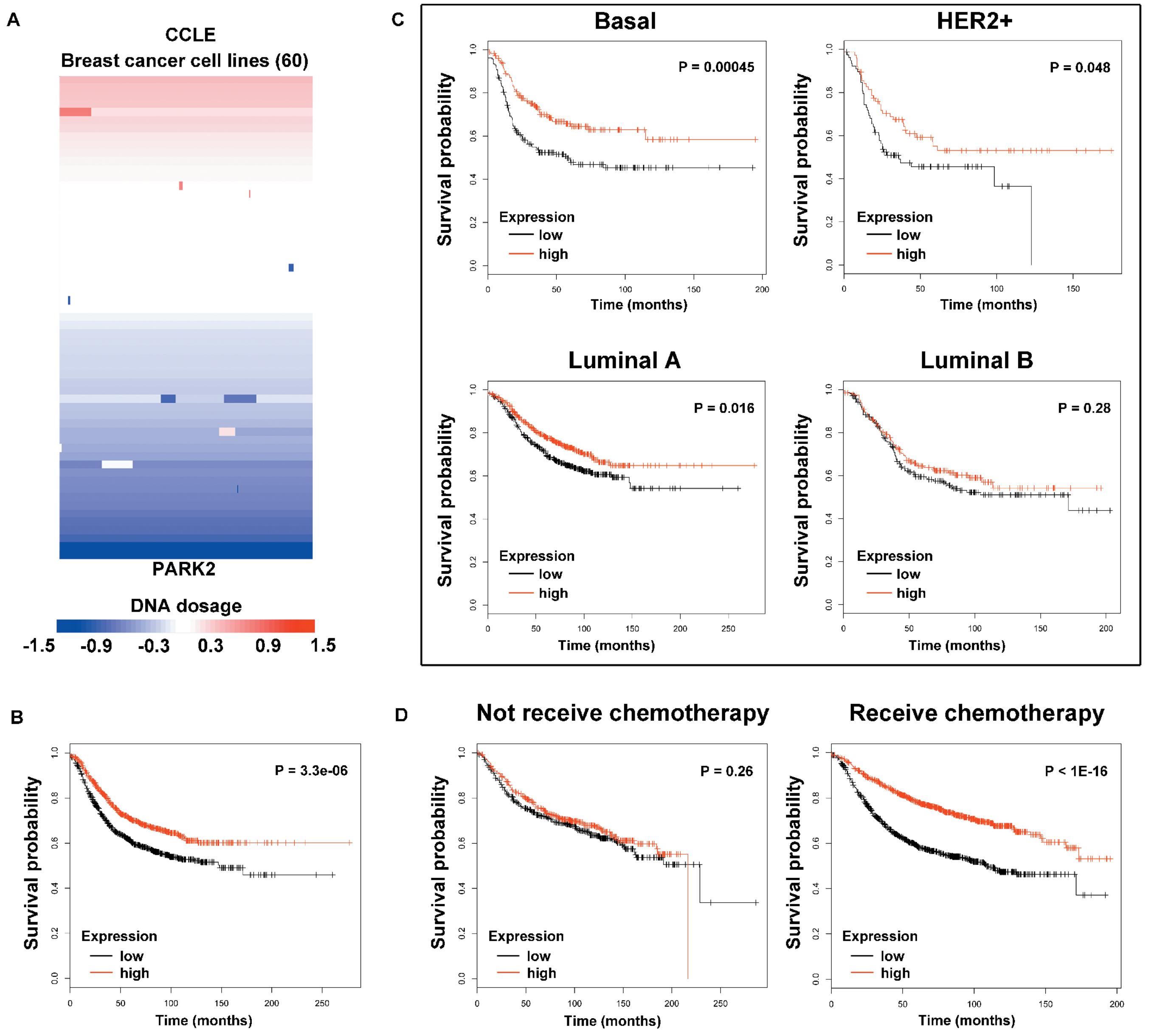

Supplementary Fig S1. Prognostic value of PARK2 expression in breast patients who received chemotherapy

A. IGV plots showing that DNA copy number loss of PARK2 occurred in 32 of 60 breast cancer cell lines.

B. Low PARK2 expression was associated with poor survival of breast cancer patients (Kaplan-Meier analysis). High expression (n=853), Low expression (n=911).

C. Low PARK2 expression was associated with poor survival of breast cancer patients within the different molecular subtypes, cohorts include basal [High expression (n=184), Low expression (n=176)], HER2+ [High expression (n=79), Low expression (n=77)], and luminal A [High expression (n=430), Low expression (n=411)]. Lower PARK2 expression was slightly correlated with poorer survival in the luminal B subtype [High expression (n=209), Low expression (n=198)].

D. Left graph: breast patients who did not receive chemotherapy, there was no difference in survival between patients with high and low expression of PARK2. High expression (n=290), Low expression (n=298). Right graph: breast patients who received chemotherapy, higher PARK2 expression was positively correlated with better survival. High expression (n=570), Low expression (n=606).

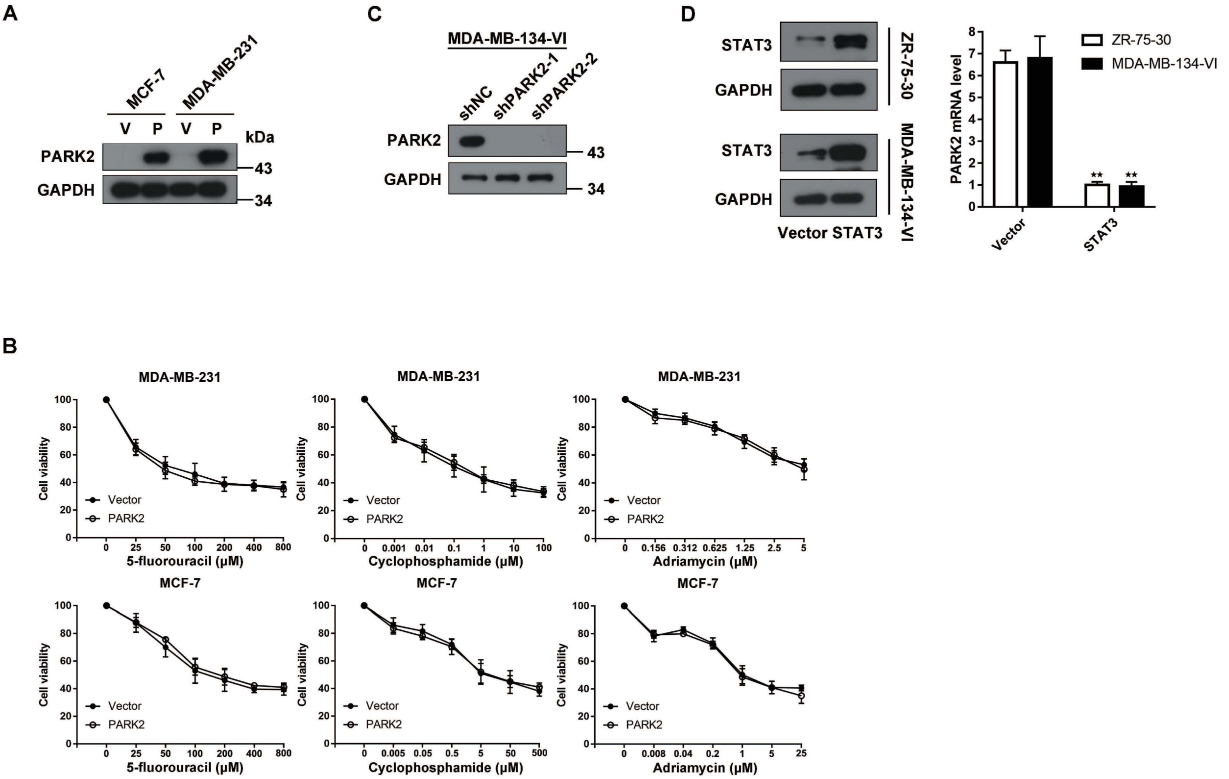

Figure S2. PARK2 overexpression does not change effectiveness of DNA-damaging drug.

A. Efficient overexpression of PARK2 in MCF-7 and MDA-MB-231 cells at translational level.

B. Cytotoxicity assays showing sensitivity to adriamycin, cyclophosphamide, and 5-fluorouracil. Breast cancer lines stably expressing either ectopic wild-type PARK2 or vector were treated with the indicated compounds for 48 h.

C. Knockdown efficiency of shRNAs for PARK2 at translational levels.

D. overexpression of STAT3 downregulated the PARK2 levels in both cell lines.

Data show mean ± s.d. N = 3. *P < 0.05, **P < 0.01

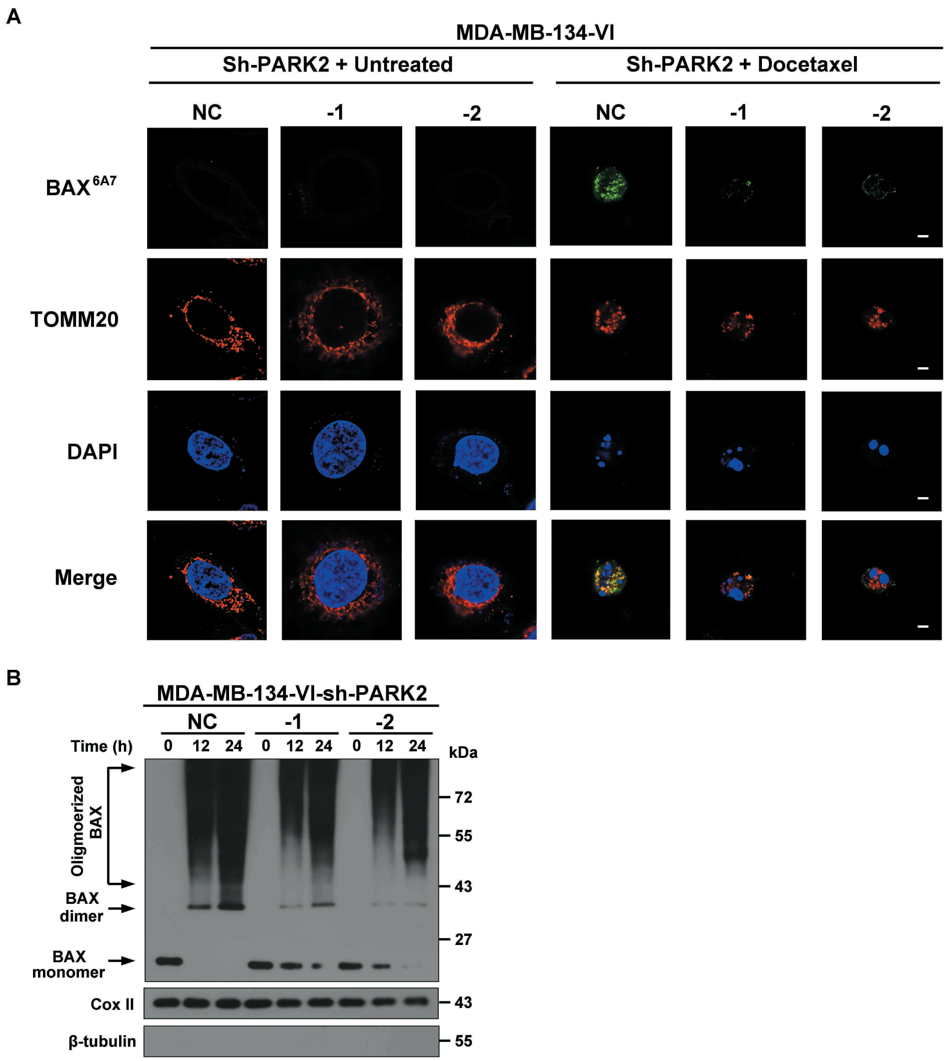

Figure S3. endogenous high expression of PARK2 was able to activate and translocate Bax to the mitochondria

A. ShRNA silencing of endogenous PARK2 resulted in significantly decrease mitochondrial translocation of BAX protein in MDA-MB-134-VI cells. Cells were analyzed by confocal microscopy. Scale bars represent 5 μm.

B. ShRNA silencing of endogenous PARK2 resulted in significantly decrease BAX oligomerization in MDA-MB-134-VI cells.

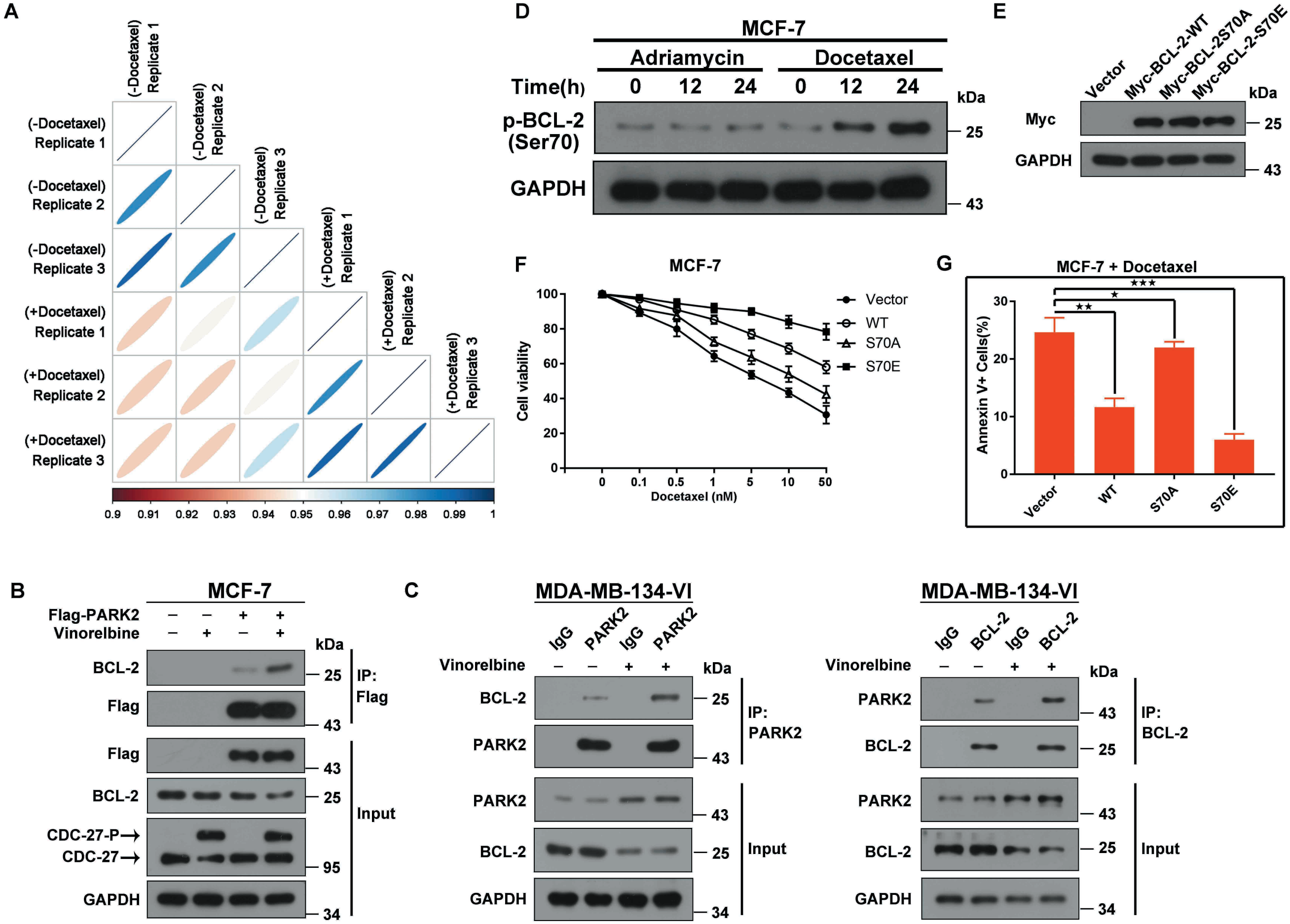

Figure S4. Vinorelbine promotes the interaction between PARK2 and BCL-2.

A. Example of experimental replicates of quantitative proteomics with Pearson correlation of 0.98 (n = 3).

B. MCF-7 cells stably expressing either ectopic wild-type PARK2 or vector (control) were treated with vinorelbine (1nM) for 24 h. Cells were lysed with RIPA buffer followed by immunoprecipitation (IP) using anti-FLAG agarose and Western blot with the indicated antibodies.

C. MDA-MB-134-VI cells were treated with vinorelbine (2nM) for 24 h. After vinorelbine treatment, co-IP was performed with endogenous PARK2 and BCL-2 in MDA-MB-134-VI cells.

D. MCF-7 cells were treated with adriamycin or docetaxel for indicated time points. Cell lysates were immunoblotted for phospho-BCL-2 (Ser70) and GAPDH.

E. Efficient overexpression of Myc-BCL-2-WT, Myc-BCL-2-S70A and Myc-BCL-2-S70E in MCF-7 at translational level.

F. Cytotoxicity assays showing sensitivity to docetaxel. MCF-7 cells stably expressing either Myc-BCL-2-WT, Myc-BCL-2-S70A or Myc-BCL-2-S70E were treated with the docetaxel for 48 h.

G. MCF-7 cells stably expressing either Myc-BCL-2-WT, Myc-BCL-2-S70A or Myc-BCL-2-S70E were treated with the docetaxel (1nM) for 48 h. Flow cytometry assay was performed.

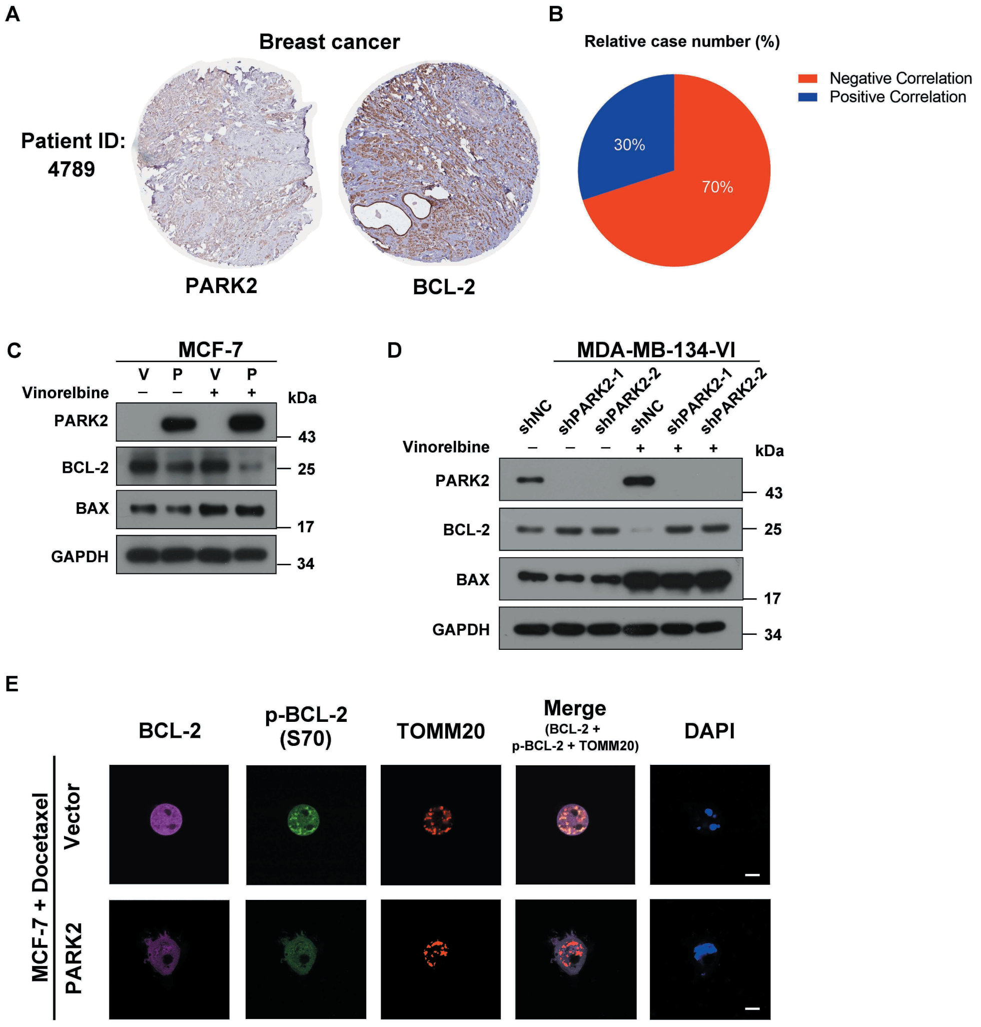

Figure S5. PARK2 promotes degradation of BCL-2.

A. Representative Immunohistochemistry images of PARK2 (Left) and BCL-2 (Right) from breast cancer patients.

B. Fan diagram. 70% of breast cancer cases have a negative correlation between PARK2 protein levels and BCL-2 protein levels (total breast cancer cases, n = 10). A&B data from The Human Protein Atlas database.

C. MCF-7 cells stably expressing either ectopic wild-type PARK2 or vector (control) were treated with vinorelbine (1nM) for 24 h and immunoblot was done.

D. MDA-MB-134-VI cells stably deleting endogenous PARK2 or NC (control) were treated with vinorelbine (2nM) for 24 h and immunoblot was done.

C-D. Cell lysates were immunoblotted for BCL-2, BAX, PARK2, and GAPDH.

E. MCF-7 cells stably expressing either ectopic wild-type PARK2 or vector (control) were treated with docetaxel (1nM) for 24 h. MCF-7 cells were co-immunostained with TOMM20 antibody (red; anti-TOMM20), BCL-2 antibody (purple; anti-BCL-2), phospho-BCL-2 (Ser70) antibody (green; anti-phospho-BCL-2 (Ser70)) and nuclei (blue; DAPI).

Cells were analyzed by confocal microscopy. Scale bars represent 5 μm.

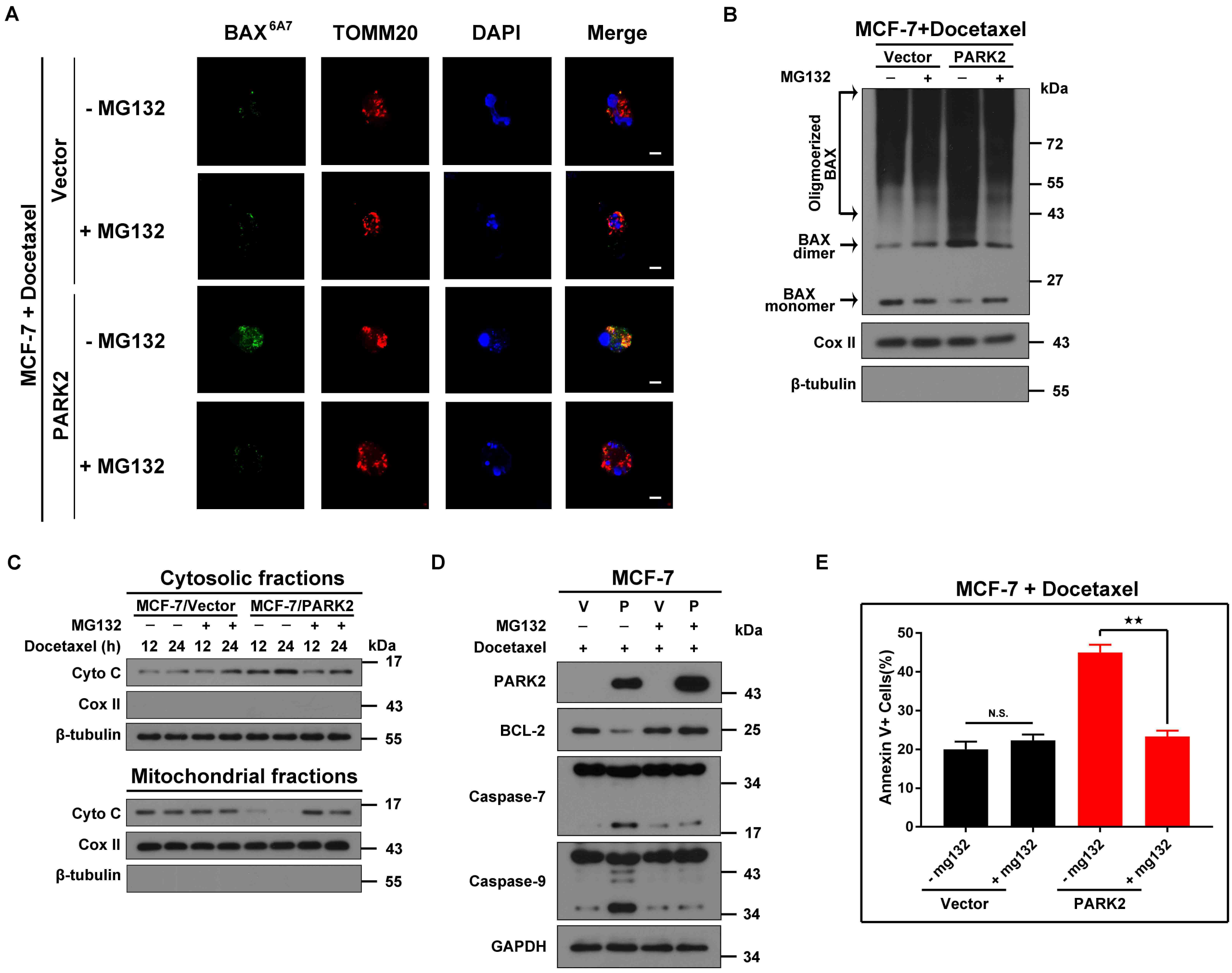

Figure S6. Under antimicrotubule drugs treatment, PARK2 was not able to induce cell death when BCL-2 is not degraded.

A. MCF-7 cells stably expressing either ectopic wild-type PARK2 or vector (control) were cultures with docetaxel (1nM) for 24 h. MCF-7 cells were then treated with MG132 (10mM) for 8h. Cells were analyzed by confocal microscopy. Scale bars represent 5 μm.

B. MCF-7 cells stably expressing either ectopic wild-type PARK2 or vector (control) were cultures with docetaxel (1nM) for 24 h. MCF-7 cells were then treated with MG132 (10mM) for 8h. After stimulation, mitochondria were fractionated. Blot was probed with anti-BAX, anti-Cox II and anti-β-tubulin.

C. MG132 inhibited the ability of PARK2 to promote mitochondrial release of cytochrome C.

D. MG132 inhibited the ability of PARK2 to activate proteolytic caspases.

E. MG132 inhibited the ability of PARK2 to induce cell death.

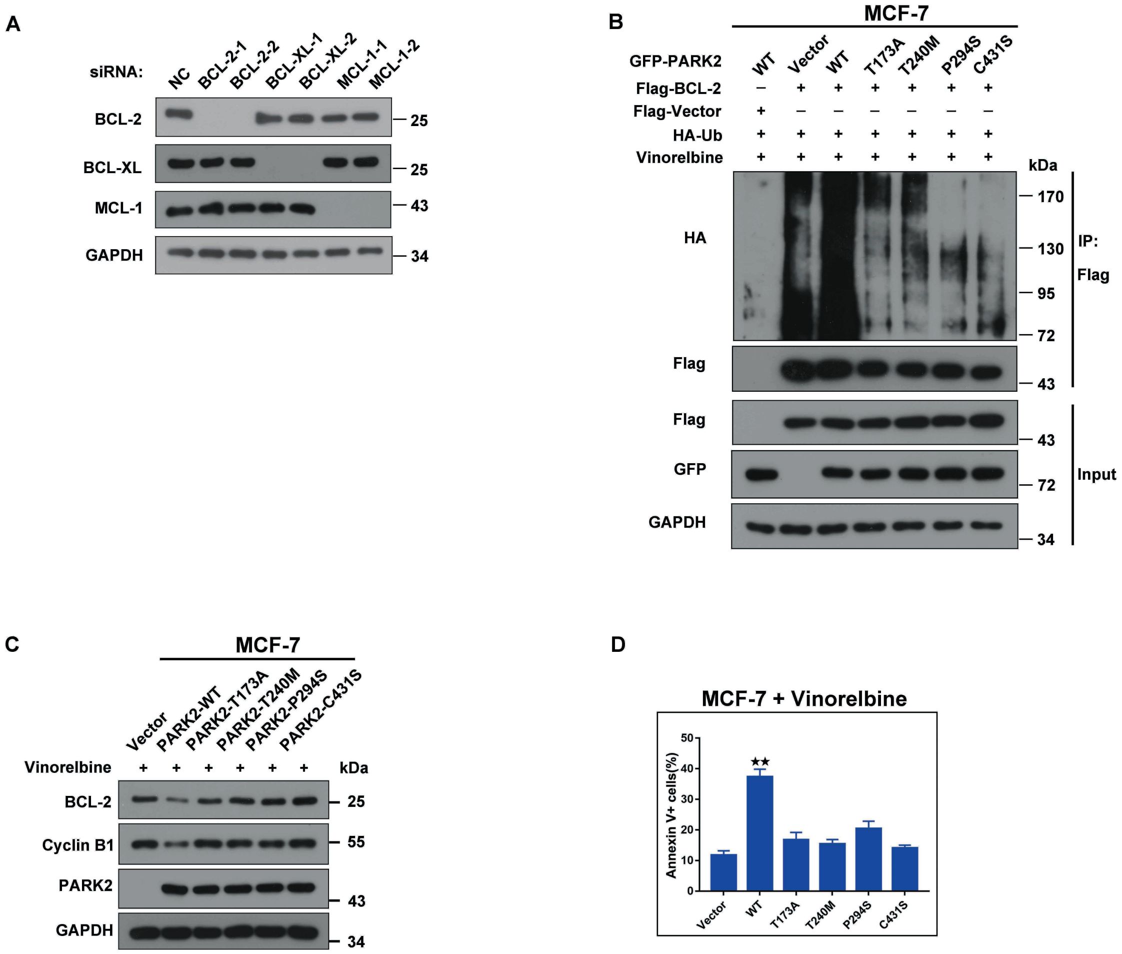

Figure S7. PARK2 loss-of-function mutants fail to enhance vinorelbine sensitivity.

A. Knockdown efficiency was analyzed by western blotting in the MCF-7 cells;

B. MCF-7 cells (Vector (control), PARK2 WT, T173, T240M, P294S, and C431S clones) were transfected with indicated vectors After transfection, cells were treated either with or without vinorelbine (1nM) for 24 h. MG132 was added 6 h prior to harvest for cell lysate. Cells were lysed with RIPA buffer followed by immunoprecipitation (IP) using anti-FLAG agarose and western blot with indicated antibody.

C. Overexpression of wild-type but not loss-of-function PARK2 mutants led to lower levels of BCL2 protein. Cyclin B1 protein was used as a positive control.

D. Overexpression of wild-type but not loss-of-function PARK2 mutants increased cellular apoptosis of breast cancer cells after vinorelbine (1nM) treatment. Data show mean ± s.d. N = 3. *P < 0.05, **P < 0.01, ***P < 0.001

Supplementary Table 1:

Compare differential proteomic changes binding to PARK2 protein before and after docetaxel treatment in the MCF-7-PARK2 cells

	Uniprot IDs	Gene	Description	Before docetaxel treatment	After docetaxel treatment	Fold change (After docetaxel treatment / Before docetaxel treatment)
1	6050	RNH1	Ribonuclease inhibitor [OS=Homo sapiens]	72	128	1.829
2	3304; 3303	HSPA1B; HSPA1A	heat shock 70 kDa protein 1A [OS=Homo sapiens]	112.3	87.7	0.796
3	1191	CLU	Clusterin [OS=Homo sapiens]	73.8	126.2	2.017
4	3312	HSPA8	Heat shock cognate 71 kDa protein [OS=Homo sapiens]	90.7	109.3	1.245
5	5591	PRKDC	DNA-dependent protein kinase catalytic subunit [OS=Homo sapiens]	100.8	99.2	1.051
6	1938	EEF2	Elongation factor 2 [OS=Homo sapiens]	97.6	102.4	1.111
7	1832	DSP	Desmoplakin [OS=Homo sapiens]	99.8	100.2	1.121
8	2023	ENO1	alpha-enolase [OS=Homo sapiens]	111.1	88.9	0.752
9	2194	FASN	Fatty acid synthase [OS=Homo sapiens]	133.7	66.3	0.611
10	6950	TCP1	T-complex protein 1 subunit alpha [OS=Homo sapiens]	95.2	104.8	1.123
11	3313	HSPA9	Stress-70 protein, mitochondrial [OS=Homo sapiens]	101	99	0.982
12	3309	HSPA5	78 kDa glucose-regulated protein [OS=Homo sapiens]	124.9	75.1	0.935
13	7431	VIM	Vimentin [OS=Homo sapiens]	103.7	96.3	0.904
14	2058	EPRS	Bifunctional glutamate/proline--tRNA ligase [OS=Homo sapiens]	120.1	79.9	0.806
15	596	BCL2	Apoptosis regulator Bcl-2 [OS=Homo sapiens]	60.6	139.4	2.406
16	6124	RPL4	60S ribosomal protein L4 [OS=Homo sapiens]	67.8	132.2	1.876
17	498	ATP5A1	ATP synthase subunit alpha, mitochondrial [OS=Homo sapiens]	97.2	102.8	1.117
18	3329	HSPD1	60 kDa heat shock protein, mitochondrial [OS=Homo sapiens]	107.6	92.4	0.879
19	8471	IRS4	insulin receptor substrate 4 [OS=Homo sapiens]	73.9	126.1	1.764
20	6188	RPS3	40S ribosomal protein S3 [OS=Homo sapiens]	93.8	106.2	1.152
21	10399	GNB2L1; RACK1	Receptor of activated protein C kinase 1 [OS=Homo sapiens]	95	105	1.102
22	5071	PARK2; PRKN	E3 ubiquitin-protein ligase parkin [OS=Homo sapiens]	139.1	130	0.935
23	5110	PCMT1	protein-L-isoaspartate(D-aspartate) O-methyltransferase [OS=Homo sapiens]	49.7	150.3	2.767
24	5052	PRDX1	peroxiredoxin-1 [OS=Homo sapiens]	67.4	132.6	1.728
8	2023	ENO1	alpha-enolase [OS=Homo sapiens]	111.1	88.9	0.752
9	2194	FASN	Fatty acid synthase [OS=Homo sapiens]	133.7	66.3	0.611
10	6950	TCP1	T-complex protein 1 subunit alpha [OS=Homo sapiens]	95.2	104.8	1.123
11	3313	HSPA9	Stress-70 protein, mitochondrial [OS=Homo sapiens]	101	99	0.982
12	3309	HSPA5	78 kDa glucose-regulated protein [OS=Homo sapiens]	124.9	75.1	0.935
13	7431	VIM	Vimentin [OS=Homo sapiens]	103.7	96.3	0.904
14	2058	EPRS	Bifunctional glutamate/proline--tRNA ligase [OS=Homo sapiens]	120.1	79.9	0.806
15	596	BCL2	Apoptosis regulator Bcl-2 [OS=Homo sapiens]	60.6	139.4	2.406
16	6124	RPL4	60S ribosomal protein L4 [OS=Homo sapiens]	67.8	132.2	1.876

17	498	ATP5A1	ATP synthase subunit alpha, mitochondrial [OS=Homo sapiens]	97.2	102.8	1.117
18	3329	HSPD1	60 kDa heat shock protein, mitochondrial [OS=Homo sapiens]	107.6	92.4	0.879
19	8471	IRS4	insulin receptor substrate 4 [OS=Homo sapiens]	73.9	126.1	1.764
20	6188	RPS3	40S ribosomal protein S3 [OS=Homo sapiens]	93.8	106.2	1.152
21	10399	GNB2L1; RACK1	Receptor of activated protein C kinase 1 [OS=Homo sapiens]	95	105	1.102
22	5071	PARK2; PRKN	E3 ubiquitin-protein ligase parkin [OS=Homo sapiens]	139.1	130	0.935
23	5110	PCMT1	protein-L-isoaspartate(D-aspartate) O-methyltransferase [OS=Homo sapiens]	49.7	150.3	2.767
24	5052	PRDX1	peroxiredoxin-1 [OS=Homo sapiens]	67.4	132.6	1.728
25	6128	RPL6	60S ribosomal protein L6 [OS=Homo sapiens]	77.1	122.9	1.654
26	8833	GMPS	GMP synthase [glutamine-hydrolyzing] [OS=Homo sapiens]	62.6	137.4	1.416
27	388697	HRNR	Hornerin [OS=Homo sapiens]	96.2	103.8	1.193
28	213	ALB	Serum albumin [OS=Homo sapiens]	118.9	81.1	0.835
29	3326	HSP90AB 1	Heat shock protein HSP 90-beta [OS=Homo sapiens]	128	72	0.671
30	3320	HSP90AA 1	Heat shock protein HSP 90-alpha [OS=Homo sapiens]	132.1	67.9	0.451
31	7317	UBA1	Ubiquitin-like modifier-activating enzyme 1 [OS=Homo sapiens]	157.7	42.3	0.416
32	226	ALDOA	fructose-bisphosphate aldolase A [OS=Homo sapiens]	158.9	41.1	0.345
33	581	BAX	Apoptosis regulator BAX [OS=Homo sapiens]	56.5	143.5	3.091
34	30968	STOML2	Stomatin-like protein 2, mitochondrial [OS=Homo sapiens]	68.3	131.7	1.76
35	6187	RPS2	40S ribosomal protein S2 [OS=Homo sapiens]	90.2	109.8	1.193
36	10575	CCT4	T-complex protein 1 subunit delta [OS=Homo sapiens]	115.5	84.5	1.005
37	6122	RPL3	60S ribosomal protein L3 [OS=Homo sapiens]	80.8	119.2	1.44
38	1915	EEF1A1	Elongation factor 1-alpha 1 [OS=Homo sapiens]	95.3	104.7	0.981
39	7203	CCT3	T-complex protein 1 subunit gamma [OS=Homo sapiens]	112.6	87.4	0.89
40	10574	CCT7	T-complex protein 1 subunit eta [OS=Homo sapiens]	88.5	111.5	0.539
41	6130	RPL7A	60S ribosomal protein L7a [OS=Homo sapiens]	65.9	134.1	2.091
42	7873	MANF	Mesencephalic astrocyte-derived neurotrophic factor [OS=Homo sapiens]	74.4	125.6	1.976
43	6129	RPL7	60S ribosomal protein L7 [OS=Homo sapiens]	72.8	127.2	1.594
44	6202	RPS8	40S ribosomal protein S8 [OS=Homo sapiens]	88.7	111.3	1.285
45	6203	RPS9	40S ribosomal protein S9 [OS=Homo sapiens]	90.2	109.8	1.203
46	6222	RPS18	40S ribosomal protein S18 [OS=Homo sapiens]	90.8	109.2	1.172
47	1973	EIF4A1	Eukaryotic initiation factor 4A-I [OS=Homo sapiens]	108.6	91.4	0.961
48	5315	PKM	Pyruvate kinase PKM [OS=Homo sapiens]	107.2	92.8	0.811
49	7184	HSP90B1	Endoplasmin [OS=Homo sapiens]	155.2	44.8	0.319
50	3945	LDHB	L-lactate dehydrogenase B chain [OS=Homo sapiens]	162.6	37.4	0.314
51	6191	RPS4X	40S ribosomal protein S4, X isoform [OS=Homo sapiens]	85.5	114.5	1.188
52	6205	RPS11	40S ribosomal protein S11 [OS=Homo sapiens]	79	121	1.145
53	3190	HNRNPK	Heterogeneous nuclear ribonucleoprotein K [OS=Homo sapiens]	100.1	99.9	1.253

54	6175	RPLP0	60S acidic ribosomal protein P0 [OS=Homo sapiens]	102.5	97.5	0.949
55	10576	CCT2	T-complex protein 1 subunit beta [OS=Homo sapiens]	76.8	123.2	0.938
56	1937	EEF1G	elongation factor 1-gamma [OS=Homo sapiens]	147.4	52.6	0.54
57	1660	DHX9	Atp-dependent rna helicase a [OS=Homo sapiens]	130.9	69.1	0.482
58	7812	CSDE1	cold shock domain-containing protein E1 [OS=Homo sapiens]	48	152	2.325
59	4670	HNRNPM	Heterogeneous nuclear ribonucleoprotein M [OS=Homo sapiens]	82.4	117.6	1.89
60	5631	PRPS1	ribose-phosphate pyrophosphokinase 1 [OS=Homo sapiens]	83.5	116.5	1.387
61	2193	FARSA	Phenylalanine--tRNA ligase alpha subunit [OS=Homo sapiens]	80.4	119.6	1.371
62	10165	SLC25A13	Calcium-binding mitochondrial carrier protein Aralar2 [OS=Homo sapiens]	67.5	132.5	1.291
63	8359;	HIST1H4A;	histone H4 [OS=Homo sapiens]	104.9	95.1	1.151
64	1434	CSE1L	Exportin-2 [OS=Homo sapiens]	144	56	0.806
65	2597	GAPDH	glyceraldehyde-3-phosphate dehydrogenase [OS=Homo sapiens]	125.1	74.9	0.64
66	908	CCT6A	T-complex protein 1 subunit zeta [OS=Homo sapiens]	140.3	59.7	0.618
67	3939	LDHA	L-lactate dehydrogenase A chain [OS=Homo sapiens]	146.3	53.7	0.396
68	3301	DNAJA1	DnaJ homolog subfamily A member 1 [OS=Homo sapiens]	74.8	125.2	1.761
69	7001	PRDX2	Peroxiredoxin-2 [OS=Homo sapiens]	69.6	130.4	1.707
70	6138	RPL15	60S ribosomal protein L15 [OS=Homo sapiens]	98.3	101.7	1.626
71	23521	RPL13A	60S ribosomal protein L13a [OS=Homo sapiens]	69.5	130.5	1.537
72	506	ATP5B	ATP synthase subunit beta, mitochondrial [OS=Homo sapiens]	79.9	120.1	1.45
73	790	CAD	CAD protein [OS=Homo sapiens]	81.4	118.6	1.344
74	7284	TUFM	elongation factor Tu, mitochondrial [OS=Homo sapiens]	89.9	110.1	1.218
75	6217	RPS16	40S ribosomal protein S16 [OS=Homo sapiens]	93.7	106.3	1.154
76	476	ATP1A1	Sodium/potassium-transporting ATPase subunit alpha-1 [OS=Homo sapiens]	106.5	93.5	1.076
77	3192	HNRNPU	Heterogeneous nuclear ribonucleoprotein U [OS=Homo sapiens]	90.1	109.9	0.97
78	6923	TCEB2; ELOB	Elongin-B [OS=Homo sapiens]	103.1	96.9	0.957
79	6189	RPS3A	40S ribosomal protein S3a [OS=Homo sapiens]	107.5	92.5	0.881
80	5358	PLS3	Plastin-3 [OS=Homo sapiens]	130.6	69.4	0.744
81	5901	RAN	GTP-binding nuclear protein RAN [OS=Homo sapiens]	119.8	80.2	0.667
82	4627	MYH9	Myosin-9 [OS=Homo sapiens]	131	69	0.627
83	47	ACLY	ATP-citrate synthase [OS=Homo sapiens]	146.8	53.2	0.536
84	5245	PHB	Prohibitin [OS=Homo sapiens]	133	67	0.523
85	7167	TPI1	Isoform 2 of Triosephosphate isomerase [OS=Homo sapiens]	151.3	48.7	0.413
86	9532	BAG2	BAG family molecular chaperone regulator 2 [OS=Homo sapiens]	56.8	143.2	2.655
87	7311	UBA52	Ubiquitin-60S ribosomal protein L40 [OS=Homo sapiens]	56.3	143.7	2.284
88	6156	RPL30	60S ribosomal protein L30 [OS=Homo sapiens]	70.5	129.5	1.776
89	4171	MCM2	DNA replication licensing factor mcm2 [OS=Homo sapiens]	69.3	130.7	1.71
90	6158	RPL28	60S ribosomal protein L28 [OS=Homo sapiens]	73.1	126.9	1.708
91	6141	RPL18	60S ribosomal protein L18 [OS=Homo sapiens]	77.1	122.9	1.457

92	5478	PPIA	peptidyl-prolyl cis-trans isomerase A [OS=Homo sapiens]	85.9	114.1	1.201
93	22948	CCT5	T-complex protein 1 subunit epsilon [OS=Homo sapiens]	98.9	101.1	1.084
94	5250	SLC25A3	Isoform B of Phosphate carrier protein, mitochondrial [OS=Homo sapiens]	90.6	109.4	1.062
95	6184	RPN1	Dolichyl-diphosphooligosaccharide--protein glycosyltransferase subunit 1 [OS=Homo sapiens]	91.5	108.5	0.979
96	26227	PHGDH	D-3-phosphoglycerate dehydrogenase [OS=Homo sapiens]	111.1	88.9	0.905
97	821	CANX	Calnexin [OS=Homo sapiens]	117.4	82.6	0.788
98	4522	MTHFD1	C-1-tetrahydrofolate synthase, cytoplasmic [OS=Homo sapiens]	144.2	55.8	0.776
99	9588	PRDX6	Peroxiredoxin-6 [OS=Homo sapiens]	117.1	82.9	0.72
100	3376	IARS	isoleucine--tRNA ligase, cytoplasmic [OS=Homo sapiens]	109.1	90.9	0.709
101	6125	RPL5	60S ribosomal protein L5 [OS=Homo sapiens]	135.3	64.7	0.533
102	142	PARP1	Poly [ADP-ribose] polymerase 1 [OS=Homo sapiens]	183.7	16.3	0.265
103	4869	NPM1	Nucleophosmin [OS=Homo sapiens]	63.9	136.1	2.055
104	6132	RPL8	60S ribosomal protein L8 [OS=Homo sapiens]	64.3	135.7	1.856
105	4736	RPL10A	60S ribosomal protein L10A [OS=Homo sapiens]	64.5	135.5	1.854
106	6142	RPL18A	60S ribosomal protein L18a [OS=Homo sapiens]	75.5	124.5	1.744
107	6137	RPL13	60S ribosomal protein L13 [OS=Homo sapiens]	74.3	125.7	1.713
108	6134	RPL10	60S ribosomal protein L10 [OS=Homo sapiens]	77.1	122.9	1.705
109	6194	RPS6	40S RIBOSOMAL PROTEIN S6 [OS=Homo sapiens]	79.4	120.6	1.496
110	4176	MCM7	DNA replication licensing factor MCM7 [OS=Homo sapiens]	83.5	116.5	1.454
111	1984	EIF5A	Eukaryotic translation initiation factor 5A-1 [OS=Homo sapiens]	69.6	130.4	1.419
112	7266	DNAJC7	DnaJ homolog subfamily C member 7 [OS=Homo sapiens]	80.9	119.1	1.41
113	6136	RPL12	60S ribosomal protein L12 [OS=Homo sapiens]	85.9	114.1	1.34
114	6193	RPS5	40S ribosomal protein S5 [OS=Homo sapiens]	89.2	110.8	1.239
115	6169	RPL38	60s ribosomal protein l38 [OS=Homo sapiens]	96.5	103.5	1.223
116	5709	PSMD3	26S proteasome non-ATPase regulatory subunit 3 [OS=Homo sapiens]	95.7	104.3	1.144
117	83858	ATAD3B	ATPase family AAA domain-containing protein 3B [OS=Homo sapiens]	94	106	1.084
118	3020; 440926; 3021	H3F3A; H3F3AP4; H3F3B	histone H3.3 [OS=Homo sapiens]	101.8	98.2	1.038
119	9524	TECR	Very-long-chain enoyl-CoA reductase [OS=Homo sapiens]	85.1	114.9	1.025
120	1654	DDX3X	ATP-dependent RNA helicase DDX3X [OS=Homo sapiens]	95.7	104.3	0.946
121	6210	RPS15A	40S ribosomal protein S15a [OS=Homo sapiens]	105.7	94.3	0.882
122	7919	DDX39B	spliceosome RNA helicase DDX39B [OS=Homo sapiens]	155.1	44.9	0.795
123	1314	COPA	coatomer subunit alpha [OS=Homo sapiens]	100.3	99.7	0.764
124	10808	HSPH1	Heat shock protein 105 kDa [OS=Homo sapiens]	119.3	80.7	0.711
125	6389	SDHA	Succinate dehydrogenase [ubiquinone] flavoprotein subunit, mitochondrial [OS=Homo sapiens]	113.4	86.6	0.673
126	5230	PGK1	phosphoglycerate kinase 1 [OS=Homo sapiens]	136.5	63.5	0.565
127	2547	XRCC6	X-ray repair cross-complementing protein 6 [OS=Homo sapiens]	131.2	68.8	0.542

128	2010	EMD	Emerin [OS=Homo sapiens]	65.2	134.8	2.185
129	26263	FBXO22	F-box only protein 22 [OS=Homo sapiens]	58.2	141.8	2.09
130	10985	GCN1L1; GCN1	eIF-2-alpha kinase activator GCN1 [OS=Homo sapiens]	43.3	156.7	1.871
131	58477	SRPRB	signal recognition particle receptor subunit beta [OS=Homo sapiens]	63.2	136.8	1.842
132	5093	PCBP1	Poly(RC)-binding protein 1 [OS=Homo sapiens]	82.3	117.7	1.765
133	4904	YBX1	Nuclease-sensitive element-binding protein 1 [OS=Homo sapiens]	82.6	117.4	1.641
134	8402	SLC25A11	Mitochondrial 2-oxoglutarate/malate carrier protein [OS=Homo sapiens]	86.7	113.3	1.575
135	10294	DNAJA2	DnaJ homolog subfamily A member 2 [OS=Homo sapiens]	71.3	128.7	1.537
136	9349	RPL23	60S ribosomal protein L23 [OS=Homo sapiens]	78.3	121.7	1.526
137	3930	LBR	Lamin-B receptor [OS=Homo sapiens]	79	121	1.512
138	6152	RPL24	60S ribosomal protein L24 [OS=Homo sapiens]	86.2	113.8	1.483
139	1072	CFL1	Cofilin-1 [OS=Homo sapiens]	83.6	116.4	1.43
140	6421	SFPQ	splicing factor, proline- and glutamine-rich [OS=Homo sapiens]	82	118	1.426
141	509	ATP5C1	ATP synthase subunit gamma, mitochondrial [OS=Homo sapiens]	81.7	118.3	1.422
142	6228	RPS23	40S ribosomal protein S23 [OS=Homo sapiens]	75.4	124.6	1.348
143	1468	SLC25A10	Mitochondrial dicarboxylate carrier [OS=Homo sapiens]	82.1	117.9	1.339
144	6164	RPL34	60S ribosomal protein L34 [OS=Homo sapiens]	87	113	1.31
145	6230	RPS25	40S ribosomal protein S25 [OS=Homo sapiens]	93.1	106.9	1.148
146	5094	PCBP2	Poly(rC)-binding protein 2 [OS=Homo sapiens]	95.5	104.5	1.125
147	203068	TUBB	tubulin beta chain [OS=Homo sapiens]	99.5	100.5	1.08
148	10642	IGF2BP1	Insulin-like growth factor 2 mRNA-binding protein 1 [OS=Homo sapiens]	111.8	88.2	0.97
149	718	C3	Complement C3 [OS=Homo sapiens]	97.7	102.3	0.866
150	448834	KPRP	Keratinocyte proline-rich protein [OS=Homo sapiens]	126.1	73.9	0.826
151	3178	HNRNPA1	Heterogeneous nuclear ribonucleoprotein A1 [OS=Homo sapiens]	108	92	0.795
152	6223	RPS19	40S ribosomal protein S19 [OS=Homo sapiens]	121.2	78.8	0.783
153	55740	ENAH	Protein enabled homolog [OS=Homo sapiens]	98.6	101.4	0.771
154	708	C1QBP	Complement component 1 Q subcomponent-binding protein, mitochondrial [OS=Homo sapiens]	120.2	79.8	0.735
155	6207	RPS13	40S ribosomal protein S13 [OS=Homo sapiens]	122.9	77.1	0.642
156	6154	RPL26	60S ribosomal protein L26 [OS=Homo sapiens]	131.4	68.6	0.61
157	2821	GPI	glucose-6-phosphate isomerase [OS=Homo sapiens]	121.7	78.3	0.488
158	1152	CKB	Creatine kinase B-type [OS=Homo sapiens]	147.9	52.1	0.431
159	5216	PFN1	profilin-1 [OS=Homo sapiens]	137.2	62.8	0.378
160	9045	RPL14	60S ribosomal protein L14 [OS=Homo sapiens]	66	134	2.102

161	6921	TCEB1; ELOC	elongin-C [OS=Homo sapiens]	71.1	128.9	1.731
162	6143	RPL19	60S ribosomal protein L19 [OS=Homo sapiens]	75.9	124.1	1.669
163	2091	FBL	rRNA 2'-O-methyltransferase fibrillarin [OS=Homo sapiens]	74.5	125.5	1.667
164	6208	RPS14	40S ribosomal protein S14 [OS=Homo sapiens]	75.7	124.3	1.629
165	292	SLC25A5	ADP/ATP translocase 2 [OS=Homo sapiens]	78.5	121.5	1.591
166	8407	TAGLN2	Transgelin-2 [OS=Homo sapiens]	79.3	120.7	1.573
167	1615	DARS	Aspartate--tRNA ligase, cytoplasmic [OS=Homo sapiens]	154.6	45.4	1.427
168	9782	MATR3	Matrin-3 [OS=Homo sapiens]	84.1	115.9	1.401
169	10061	ABCF2	ATP-binding cassette sub-family F member 2 [OS=Homo sapiens]	77.3	122.7	1.366
170	6566	SLC16A1	Monocarboxylate transporter 1 [OS=Homo sapiens]	81.6	118.4	1.356
171	6157	RPL27A	60S ribosomal protein L27a [OS=Homo sapiens]	84.2	115.8	1.331
172	6206	RPS12	40S ribosomal protein S12 [OS=Homo sapiens]	87.1	112.9	1.321
173	10606	PAICS	multifunctional protein ADE2 [OS=Homo sapiens]	80.3	119.7	1.317
174	55750	AGK	Acylglycerol kinase, mitochondrial [OS=Homo sapiens]	78.4	121.6	1.243
175	6139	RPL17	60S ribosomal protein L17 [OS=Homo sapiens]	76.1	123.9	1.231
176	6224	RPS20	40S ribosomal protein S20 [OS=Homo sapiens]	97.3	102.7	1.22
177	2618	GART	trifunctional purine biosynthetic protein adenosine-3 [OS=Homo sapiens]	91.3	108.7	1.146
178	3187	HNRNPH1	Heterogeneous nuclear ribonucleoprotein H [OS=Homo sapiens]	131.1	68.9	1.145
179	3183	HNRNPC	Heterogeneous nuclear ribonucleoproteins C1/C2 [OS=Homo sapiens]	103	97	1.011
180	4172	MCM3	DNA replication licensing factor mcm3 [OS=Homo sapiens]	95.2	104.8	1.006
181	10155	TRIM28	Transcription intermediary factor 1-beta [OS=Homo sapiens]	97.5	102.5	0.969
182	3921	RPSA	40S ribosomal protein SA [OS=Homo sapiens]	59.4	140.6	0.941
183	26986	PABPC1	Polyadenylate-binding protein 1 [OS=Homo sapiens]	78.8	121.2	0.938
184	5917	RARS	arginine--tRNA ligase, cytoplasmic [OS=Homo sapiens]	105.8	94.2	0.928
185	7965	AIMP2	aminoacyl tRNA synthase complex-interacting multifunctional protein 2 [OS=Homo sapiens]	96	104	0.914
186	23524	SRRM2	serine/arginine repetitive matrix protein 2 [OS=Homo sapiens]	101.4	98.6	0.913
187	60	ACTB	Actin, cytoplasmic 1 [OS=Homo sapiens]	113.9	86.1	0.797
188	6897	TARS	Threonine--tRNA ligase, cytoplasmic [OS=Homo sapiens]	124.3	75.7	0.779
189	10971	YWHAQ	14-3-3 protein theta [OS=Homo sapiens]	108.3	91.7	0.744
190	5223; 643576	PGAM1; LOC643576	Phosphoglycerate mutase 1 [OS=Homo sapiens]	118.7	81.3	0.738
191	2617	GARS	Glycine--tRNA ligase [OS=Homo sapiens]	68.5	131.5	0.707
192	10130	PDIA6	Protein disulfide-isomerase A6 [OS=Homo sapiens]	123.7	76.3	0.566
193	760	CA2	Carbonic anhydrase 2 [OS=Homo sapiens]	124.5	75.5	0.563
194	8661	EIF3A	Eukaryotic translation initiation factor 3 subunit A [OS=Homo sapiens]	151.7	48.3	0.506

195	3007	HIST1H1D	Histone H1.3 [OS=Homo sapiens]	144.8	55.2	0.365
196	6201	RPS7	40S ribosomal protein S7 [OS=Homo sapiens]	154.7	45.3	0.323
197	6917	TCEA1	Transcription elongation factor A protein 1 [OS=Homo sapiens]	46.6	153.4	2.772
198	23197	FAF2	FAS-associated factor 2 [OS=Homo sapiens]	43.8	156.2	2.704
199	9688	NUP93	Nuclear pore complex protein Nup93 [OS=Homo sapiens]	57.2	142.8	1.836
200	5111	PCNA	proliferating cell nuclear antigen [OS=Homo sapiens]	142.1	57.9	1.597
201	6165	RPL35A	60S ribosomal protein L35a [OS=Homo sapiens]	74	126	1.585
202	60496	AASDHPPT	L-aminoadipate-semialdehyde dehydrogenase-phosphopantetheinyl transferase [OS=Homo sapiens]	79	121	1.537
203	11160	ERLIN2	Erlin-2 [OS=Homo sapiens]	81.2	118.8	1.505
204	8813	DPM1	Dolichol-phosphate mannosyltransferase subunit 1 [OS=Homo sapiens]	77.2	122.8	1.501
205	11034	DSTN	Destrin [OS=Homo sapiens]	69.5	130.5	1.49
206	9774	BCLAF1	Bcl-2-associated transcription factor 1 [OS=Homo sapiens]	87.3	112.7	1.475
207	26135	SERBP1	Plasminogen activator inhibitor 1 RNA-binding protein [OS=Homo sapiens]	78.6	121.4	1.454
208	9584	RBM39	RNA-binding protein 39 [OS=Homo sapiens]	82.1	117.9	1.447
209	52	ACP1	Low molecular weight phosphotyrosine protein phosphatase [OS=Homo sapiens]	83	117	1.432
210	6232	RPS27	40S ribosomal protein S27 [OS=Homo sapiens]	79.6	120.4	1.428
211	8721	EDF1	Endothelial differentiation-related factor 1 [OS=Homo sapiens]	82.9	117.1	1.421
212	6173	RPL36A	60S ribosomal protein L36a [OS=Homo sapiens]	78.2	121.8	1.41
213	51441	YTHDF2	YTH domain-containing family protein 2 [OS=Homo sapiens]	83.1	116.9	1.364
214	6144	RPL21	60S ribosomal protein L21 [OS=Homo sapiens]	82.4	117.6	1.358
215	4144	MAT2A	S-adenosylmethionine synthase isoform type-2 [OS=Homo sapiens]	74.4	125.6	1.352
216	10856	RUVBL2	RuvB-like 2 [OS=Homo sapiens]	93.3	106.7	1.222
217	6135	RPL11	60S ribosomal protein L11 [OS=Homo sapiens]	89.4	110.6	1.207
218	51495	PTPLAD1; HACD3	Very-long-chain (3R)-3-hydroxyacyl-CoA dehydratase 3 [OS=Homo sapiens]	87.4	112.6	1.147
219	388698	FLG2	Filaggrin-2 [OS=Homo sapiens]	101.1	98.9	1.135
220	6204	RPS10	40S ribosomal protein S10 [OS=Homo sapiens]	88.9	111.1	1.131
221	6231; 101929876; 728937	RPS26; LOC101929876; RPS26P25	40S ribosomal protein S26 [OS=Homo sapiens]	91.1	108.9	1.112
222	10432; 100526737	RBM14; RBM14-RBM4	RNA-binding protein 14 [OS=Homo sapiens]	63.6	136.4	1.085
223	1828	DSG1	Desmoglein-1 [OS=Homo sapiens]	142.2	57.8	1.058
224	377	ARF3	ADP-ribosylation factor 3 [OS=Homo sapiens]	106.7	93.3	0.994
225	5705	PSMC5	26S proteasome regulatory subunit 8 [OS=Homo sapiens]	102.4	97.6	0.991

226	1655	DDX5	probable ATP-dependent RNA helicase DDX5 [OS=Homo sapiens]	98.9	101.1	0.983
227	10521	DDX17	Isoform 2 of Probable ATP-dependent RNA helicase DDX17 [OS=Homo sapiens]	105.2	94.8	0.919
228	197	AHSG	Alpha-2-HS-glycoprotein [OS=Homo sapiens]	106.9	93.1	0.889
229	5725	PTBP1	Polypyrimidine tract-binding protein 1 [OS=Homo sapiens]	110.8	89.2	0.85
230	293	SLC25A6	ADP/ATP translocase 3 [OS=Homo sapiens]	74.4	125.6	0.714
231	3181	HNRNPA 2B1	heterogeneous nuclear ribonucleoproteins A2/B1 [OS=Homo sapiens]	147.4	52.6	0.672
232	7531	YWHAE	14-3-3 protein epsilon [OS=Homo sapiens]	131.8	68.2	0.5
233	29789	OLA1	obg-like ATPase 1 [OS=Homo sapiens]	142.5	57.5	0.493
234	2287	FKBP3	peptidyl-prolyl cis-trans isomerase FKBP3 [OS=Homo sapiens]	61.3	138.7	2.263
235	4150	MAZ	Myc-associated zinc finger protein [OS=Homo sapiens]	59.9	140.1	2.235
236	6176	RPLP1	60S acidic ribosomal protein P1 [OS=Homo sapiens]	51	149	2.133
237	3106	HLA-B	HLA class I histocompatibility antigen, B-7 alpha chain [OS=Homo sapiens]	71	129	1.851
238	6161	RPL32	60S ribosomal protein L32 [OS=Homo sapiens]	52.1	147.9	1.839
239	6168	RPL37A	60S ribosomal protein L37a [OS=Homo sapiens]	73.1	126.9	1.756
240	6155	RPL27	60S ribosomal protein L27 [OS=Homo sapiens]	70.6	129.4	1.7
241	79751	SLC25A2 2	Mitochondrial glutamate carrier 1 [OS=Homo sapiens]	79.1	120.9	1.658
242	26354	GNL3	Guanine nucleotide-binding protein-like 3 [OS=Homo sapiens]	75.7	124.3	1.594
243	7009	TMBIM6	Bax inhibitor 1 [OS=Homo sapiens]	75.4	124.6	1.587
244	7417	VDAC2	Voltage-dependent anion-selective channel protein 2 [OS=Homo sapiens]	85.1	114.9	1.531
245	6628	SNRPB	Small nuclear ribonucleoprotein-associated proteins B and B' [OS=Homo sapiens]	76.6	123.4	1.522
246	7295	TXN	thioredoxin [OS=Homo sapiens]	74.9	125.1	1.515
247	117159	DCD	Dermcidin [OS=Homo sapiens]	79.5	120.5	1.421
248	55327	LIN7C	Protein lin-7 homolog C [OS=Homo sapiens]	89.2	110.8	1.285
249	54927	CHCHD3	MICOS complex subunit MIC19 [OS=Homo sapiens]	89.9	110.1	1.188
250	8349	HIST2H2 BE	Histone H2B type 2-E [OS=Homo sapiens]	104.9	95.1	1.083
251	10376	TUBA1B	Tubulin alpha-1B chain [OS=Homo sapiens]	100.5	99.5	1.058
252	23020	SNRNP20 0	U5 small nuclear ribonucleoprotein 200 kDa helicase [OS=Homo sapiens]	92.4	107.6	1.049
253	3040; 3039	HBA2; HBA1	Hemoglobin subunit alpha [OS=Homo sapiens]	108.7	91.3	0.987
254	10018	BCL2L11	Bcl-2-like protein 11 [OS=Homo sapiens]	100.8	99.2	0.985
255	5518	PPP2R1A	serine/threonine-protein phosphatase 2A 65 kDa regulatory subunit A alpha isoform [OS=Homo sapiens]	98.7	101.3	0.884
256	4057	LTF	Lactotransferrin [OS=Homo sapiens]	109.5	90.5	0.838
257	22820	COPG1	Coatomer subunit gamma-1 [OS=Homo sapiens]	115.7	84.3	0.729

258	440689	HIST2H2 BF	Histone H2B type 2-F [OS=Homo sapiens]	115.5	84.5	0.719
259	10383	TUBB4B	Tubulin beta-4B chain [OS=Homo sapiens]	110.2	89.8	0.717
260	7178	TPT1	Translationally-controlled tumor protein [OS=Homo sapiens]	118.5	81.5	0.695
261	3295	HSD17B4	peroxisomal multifunctional enzyme type 2 [OS=Homo sapiens]	123.6	76.4	0.618
262	11224	RPL35	60S ribosomal protein L35 [OS=Homo sapiens]	132	68	0.512
263	7534	YWHAZ	14-3-3 protein zeta/delta [OS=Homo sapiens]	138.7	61.3	0.502
264	3843	IPO5	Importin-5 [OS=Homo sapiens]	132.6	67.4	0.499
265	6133	RPL9	60S ribosomal protein L9 [OS=Homo sapiens]	137.7	62.3	0.466
266	6279	S100A8	Protein S100-A8 [OS=Homo sapiens]	58.7	141.3	2.512
267	23435	TARDBP	TAR DNA-binding protein 43 [OS=Homo sapiens]	60.6	139.4	2.302
268	5319	PLA2G1B	Phospholipase A2 [OS=Homo sapiens]	63.5	136.5	2.149
269	6159	RPL29	60S ribosomal protein L29 [OS=Homo sapiens]	61.7	138.3	1.907
270	5644	PRSS1	Trypsin-1 [OS=Homo sapiens]	68	132	1.825
271	7448	VTN	Vitronectin [OS=Homo sapiens]	69	131	1.776
272	84284	NTPCR	Cancer-related nucleoside-triphosphatase [OS=Homo sapiens]	76.4	123.6	1.617
273	9538	EI24	Etoposide-induced protein 2.4 homolog [OS=Homo sapiens]	89.9	110.1	1.354
274	4666	NACA	Nascent polypeptide-associated complex subunit alpha [OS=Homo sapiens]	89.4	110.6	1.344
275	4282	MIF	Macrophage Migration inhibitory factor [OS=Homo sapiens]	79.9	120.1	1.332
276	8338	HIST2H2 AC	Histone H2A type 2-C [OS=Homo sapiens]	86.2	113.8	1.319
277	10726	NUDC	nuclear migration protein nudC [OS=Homo sapiens]	89.1	110.9	1.245
278	94239	H2AFV	Histone H2A.V [OS=Homo sapiens]	96.7	103.3	1.233
279	54205	CYCS	cytochrome c [OS=Homo sapiens]	96.1	103.9	1.14
280	6836	SURF4	surfeit locus protein 4 [OS=Homo sapiens]	102.9	97.1	1.087
281	221613	HIST1H2 AA	Histone H2A type 1-A [OS=Homo sapiens]	91	109	1.065
282	2926	GRSF1	G-rich sequence factor 1 [OS=Homo sapiens]	101.3	98.7	0.974
283	8667	EIF3H	Eukaryotic translation initiation factor 3 subunit H [OS=Homo sapiens]	98.4	101.6	0.951
284	7846	TUBA1A	tubulin alpha-1A chain [OS=Homo sapiens]	103.1	96.9	0.939
285	10797	MTHFD2	Bifunctional methylenetetrahydrofolate dehydrogenase/cyclohydrolase, mitochondrial [OS=Homo sapiens]	104.3	95.7	0.917
286	10598	AHSA1	activator of 90 kDa heat shock protein ATPase homolog 1 [OS=Homo sapiens]	107.7	92.3	0.838
287	6147	RPL23A	60S ribosomal protein L23a [OS=Homo sapiens]	111.9	88.1	0.787
288	51631	LUC7L2	Putative RNA-binding protein Luc7-like 2 [OS=Homo sapiens]	115.7	84.3	0.766
289	2638	GC	vitamin D-binding protein [OS=Homo sapiens]	114.2	85.8	0.752
290	6160	RPL31	60S ribosomal protein L31 [OS=Homo sapiens]	143.2	56.8	0.397

Supplementary Table 2	
ANTIBODY and REAGENT	COMPANY and CODE
PARK2	CST, PRK8
PARK2	Abcam, ab15494
PARK2	Santa Cruz, sc-32282
GAPDH	TransGen Biotechnology, HC301-02
BCL-2	Santa Cruz, sc-7382
BCL-2	CST, #15071
Phospho-Bcl-2 (Ser70)	CST, #9139
STAT3	CST, #2827
Bax	Santa Cruz, sc-7480
Caspase-7	CST, #9494
Caspase-9	CST, #9502
PARP	CST, #9532
Bax 6A7	BD, 556467
Myc	CST, #2272
Flag	CST, #8146
Cyclin D1	CST, #2922
Cyclin B1	CST, #4138
CDC27	Santa Cruz, sc-9972
HA	CST, #3724
GFP	CST, #2555
Docetaxel	Selleck, S1148
Vinorelbine	Selleck, S4269
Adriamycin	Selleck, S1208
Cyclophosphamide	Selleck, S2057
Fluorouracil	Selleck, S1209
MG-132	Selleck, S2619
Cycloheximide	Sigma
Doxycycline	Selleck, S4163

The following oligonucleotides for shRNA (5’- -3’)
Human PARK2 shRNA 1
F: CCGGCGTGATTTGCTTAGACTGTTTCTCGAGAAACAGTCTAAGCAAATCACGTTTTTG
R: AATTCAAAAACGTGATTTGCTTAGACTGTTTCTCGAGAAACAGTCTAAGCAAATCACG

Human PARK2 shRNA 2
F: CCGGCTTAGACTGTTTCCACTTATACTCGAGTATAAGTGGAAACAGTCTAAGTTTTTG
R: AATTCAAAAACTTAGACTGTTTCCACTTATACTCGAGTATAAGTGGAAACAGTCTAAG

qPCR Primers-PARK2 (5’- -3’) F: ACAAGACTCAATGATCGGCAG
qPCR Primers-PARK2 (5’- -3’) R: TTCTTTACATTCCCGGCAGA

Chip-qPCR Primers-PARK2 (5’- -3’) F: ATCCCCATTGTCCCATCACCCT
Chip-qPCR Primers-PARK2 (5’- -3’) R: ATGAGGACGTTGCTTACTTCGCTGAG

The following oligonucleotides for siRNA (5’- -3’)

Human STAT3 siRNA 1	GCACAATCTACGAAGAATCAA
Human STAT3 siRNA 2	GCAAAGAATCACATGCCACTT
Human BCL-2 siRNA 1	GGGAGAUAGUGAUGAAGUA
Human BCL-2 siRNA 2	GGAUCAUGCUGUACUUAAA
Human BCL-XL siRNA 1	GGUAUGGAAGGGUUUGUGG
Human BCL-XL siRNA 2	GGAGACUAGAUUGCCUUUG
Human MCL-1 siRNA 1	GGACUUUUAUACCUGUUAU
Human MCL-1 siRNA 2	GGACACAAAGCCAAUGGGC
